# Loss of PGC-1α in RPE induces mesenchymal transition and promotes retinal degeneration

**DOI:** 10.26508/lsa.201800212

**Published:** 2019-05-17

**Authors:** Mariana Aparecida Brunini Rosales, Daisy Y Shu, Jared Iacovelli, Magali Saint-Geniez

**Affiliations:** 1 Schepens Eye Research Institute of Massachusetts Eye and Ear, Boston, MA; 2 Department of Ophthalmology, Harvard Medical School, Boston, MA

## Abstract

The retinal pigment epithelium (RPE) supports visual processing and photoreceptor homeostasis via energetically demanding cellular functions. Here, we describe the consequences of repressing peroxisome proliferator-activated receptor γ coactivator-1 α (PGC-1α), a master regulator of mitochondrial function and biogenesis, on RPE epithelial integrity. The sustained silencing of PGC-1α in differentiating human RPE cells affected mitochondria/autophagy function, redox state, and impaired energy sensor activity ultimately inducing epithelial to mesenchymal transition (EMT). Adult conditional knockout of PGC-1 coactivators in mice resulted in rapid RPE dysfunction and transdifferentiation associated with severe photoreceptor degeneration. RPE anomalies were characteristic of autophagic defect and mesenchymal transition comparable with the ones observed in age-related macular degeneration. These findings demonstrate that PGC-1α is required to maintain the functional and phenotypic status of RPE by supporting the cells’ oxidative metabolism and autophagy-mediated repression of EMT.

## Abstract (precis)

Sustained loss of PGC-1α in RPE cells triggers mitochondrial/autophagic dysfunction and oxidative damage resulting in epithelial dedifferentiation and mesenchymal transition. RPE dysfunction caused by deletion of the PGC-1 coactivators in vivo causes retinal degeneration.

## Introduction

The retina pigment epithelium (RPE) is a highly specialized monolayer of pigmented cuboidal cells separating the neural retina from the choroidal vasculature and serving critical roles to maintain retinal homeostasis and visual function. Integrity of the RPE epithelial phenotype is crucial for the cells to execute their functions, which include visual pigment recycling, photoreceptor outer segment (POS) phagocytosis, secretion of neurotrophic factors and cytokines, light absorption, ion and water transport, and maintenance of the outer blood–retinal barrier (Strauss, 2005).

Like most postmitotic cells, RPE functional maturation depends on the induction of a specific metabolic program able to support their energetic requirements. In vitro maturation of human RPE cells was found to be correlated with increased mitochondrial biogenesis and oxidative metabolism (Adijanto & Philp, 2014; Iacovelli et al, 2016). Establishment of this metabolic program appears to be, at least in part, controlled by peroxisome proliferator–activated receptor γ coactivator 1α (PGC-1α), a master regulator of mitochondrial biogenesis and function (Wu et al, 1999; Fernandez-Marcos & Auwerx, 2011), whose expression in RPE increases in alignment with their metabolic and functional maturation (Iacovelli et al, 2016). Moreover, PGC-1α gain-of-function was shown to enhance RPE metabolic functions and resistance to oxidative stress through the induction of oxidative metabolic genes and antioxidant enzymes (Iacovelli et al, 2016). Additional evidence that oxidative metabolism is essential for the maintenance of RPE functional and morphological integrity comes from recent clinicopathologic analysis of age-related macular degeneration (AMD). AMD, in particular its dry form, is characterized by focal RPE dysfunction and/or atrophy leading to the degeneration of the overlying photoreceptor and focal vision loss. Decreased expression or signaling of key components of the RPE antioxidant mechanism and increase in mitochondrial DNA damage from oxidative stress have been described in aged and AMD eyes (Lin et al, 2011; Golestaneh et al, 2016). Furthermore, mitochondrial defects and increased sensitivity of oxidative damage associated with PGC-1α repression were also observed in iPS-derived RPE cells from dry AMD patients (Golestaneh et al, 2016), suggesting that RPE metabolic alteration and oxidative damage act as key initiating events in the activation of the various degenerative mechanisms associated with AMD pathogenesis. Because PGC-1α is a major regulator of mitochondrial function, it may also be an important player in AMD pathogenesis (Kaarniranta et al, 2018) and global knockout mouse models for PGC-1α have been found to mimic some of the degenerative processes characteristic of human AMD (Egger et al, 2012; Zhang et al, 2018). However, a recent study using cultures of primary RPE cells from AMD donors provided conflicting results as diseased cells were found to express higher levels of PGC-1α and to be more resistant to oxidative stress (Ferrington et al, 2017). Although a direct implication for PGC-1α in AMD pathogenesis remains to be determined, it is clear that its expression and function are tightly correlated with RPE viability and function.

The interplay between cellular metabolism and autophagy is emerging as a critical regulatory mechanism for maintaining epithelial cell integrity and actively repressing epithelial to mesenchymal transition (EMT) (Gugnoni et al, 2016; Morandi et al, 2017).

EMT of RPE cells is central to numerous degenerative ocular diseases, including proliferative vitreoretinopathy (PVR) (Machemer et al, 1978; Casaroli–Marano et al, 1999) and AMD (Hirasawa et al, 2011). It is well established that mitochondrial dysfunction can drive epithelial cell degeneration and EMT through down-regulation of oxidative metabolism, increase of ROS production, and mtDNA damage (Guerra et al, 2017). Autophagy, which is controlled by the AMPK/mTOR energy sensor signaling axis, can also directly regulate EMT molecular reprogramming by selective degradation of major EMT molecular switches (Qiang et al, 2014; Bertrand et al, 2015; Grassi et al, 2015).

As a key regulator of oxidative metabolism and mitochondrial health, PGC-1α is likely involved in controlling RPE autophagic function as shown in skeletal muscle cells (Vainshtein et al, 2015). Indeed, PGC-1α was found to be transcriptionally induced to support POS phagocytosis (Roggia & Ueta, 2015). To further determine the critical contributions of PGC-1α in coordinating both energy metabolism and epithelial phenotypic features of RPE, we examined the in vitro and in vivo consequences of PGC-1α deletion on RPE metabolic, autophagic, and epithelial status.

## Results

### PGC-1α repression causes RPE mitochondrial dysfunction and oxidative stress

**Figure S1.**
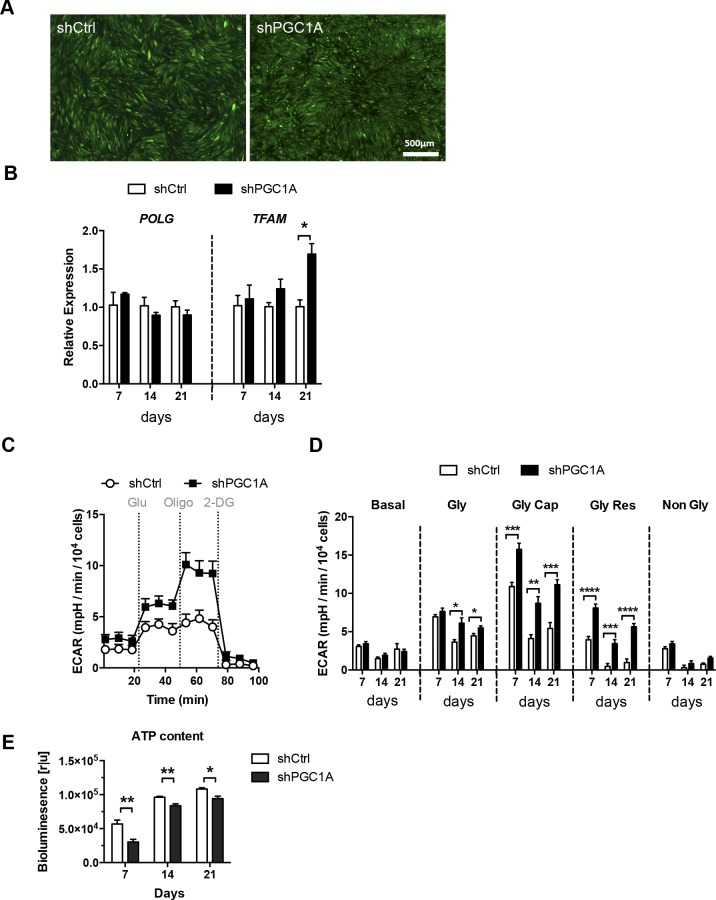
Effect of PGC-1α silencing on RPE mitochondrial replication genes and glycolytic function. **(A)** Live cell imaging of FACS sorted GFP+ shPGC1A and shCtrl ARPE-19 cells confirming the homogeneity of the cell lines. **(B)** Longitudinal gene expression analysis of the mitochondrial DNA polymerase γ, POLG, and the mitochondrial transcription factor A, TFAM. **(C)** Glycolytic function measured by ECAR of shCtrl and shPGC1A cells matured for 14 d, after sequential injections of glucose (Glu), oligomycin (Oligo), and 2-deoxyglucose (2-DG). **(D)** ECAR profile of shCtrl and shPGC1A cells at days 7, 14, and 21 of maturation showing basal ECAR levels (Basal), glycolytic rate (Gly), maximum glycolytic capacity (Gly Cap), glycolytic reserve (Gly Res), and nonglycolytic acidification (Non Gly) (n = 6–12). **(E)** Quantification of cytoplasmic ATP levels in shCtrl and shPGC1A cells at days 7, 14, and 21 (n = 4). Error bars are means ± SEM. Data were analyzed by multiple unpaired t-test comparisons using the Holm–Sidak method. *P ≤ 0.05; **P ≤ 0.01; ***P ≤ 0.001; ****P ≤ 0.0001 compared with their respective shCtrl at each time points.

**Figure 1.**
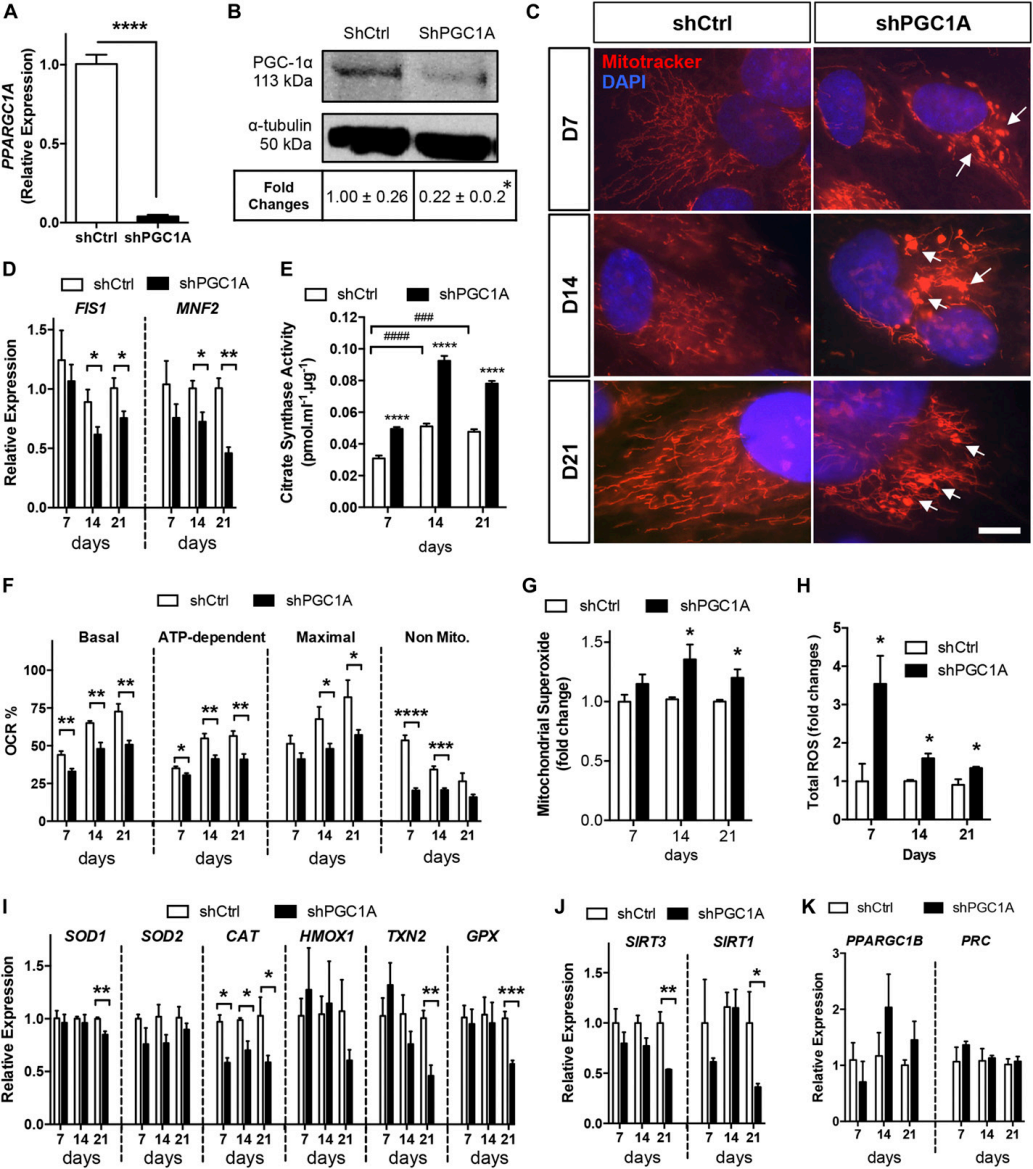
PGC-1α silencing in RPE cells promotes mitochondrial dysfunction and oxidative stress. **(A, B)** Validation of PGC-1α silencing at the mRNA and protein levels by quantitative RT-PCR (A) and Western blotting (B) in ARPE-19 cells matured for 7 d (n = 3–4 per condition, respectively). **(C)** Evaluation of mitochondrial morphology using MitoTracker (red) and DAPI (nuclei, blue) co-staining showing prominent shape changes from tubular to “donut-blob” formation in shPGC1A cells (arrows). Scale bar is 10 μm. **(D)** Quantification of the mitochondrial dynamics genes FIS1 and MFN2 by qPCR (n = 3–4). **(E)** CSA quantification (n = 4). **(F)** OCR parameter measurements of shPGC1A and shCtrl ARPE-19 at day 7, 14, and 21 (n = 5). **(G, H)** Quantification of mitochondrial superoxide production using MitoSox Red (n = 5–7) (G) and cytoplasmic ROS content with DCFH-DA (n = 3–6) (H). **(I)** Relative expression of antioxidant enzyme genes—catalase (CAT), cytosolic superoxide dismutase (SOD1), mitochondrial thioredoxin (TXN2), and glutathione peroxidase (GPX)—analyzed by qPCR (n = 3–4). **(J)** Sirtuins (SIRT) enzymes 1 and 3 gene expression analysis by qPCR (n = 3–4). **(K)** PGC-1 isoform gene expression, PGC-1β, and PRC (n = 3). Error bars are means ± SEM. Data were analyzed by multiple unpaired *t* test comparisons using the Holm–Sidak method (A–D, F–K) or ANOVA followed by Tukey’s multiple comparison test (E). *P ≤ 0.05; **P ≤ 0.01; ***P ≤ 0.001; ****P ≤ 0.0001 compared with their respective shCtrl at each time points. ^###^*P* ≤ 0.001; ^####^*P* ≤ 0.0001 compared with day 7 shCtrl.

To investigate the role of PGC-1α in RPE metabolism, integrity, and function, we silenced PGC-1α in the human RPE cell line ARPE-19 by lentivirus-mediated delivery of shRNA. Efficient repression of PGC-1α expression in GFP+ ARPE-19 cells (Fig S1A) was confirmed at the mRNA (≈96% loss, Fig 1A) and protein levels (≈78% loss, Fig 1B) after in vitro maturation for 7 d, the time of maximal PGC-1α induction in differentiating parental cells (Iacovelli et al, 2016). First, we evaluated the effect of silencing PGC-1α on mitochondrial morphology and metabolic function in shPGC-1α ARPE-19 cells. MitoTracker staining in cells matured for 7, 14, and 21 d showed severe disorganization of the mitochondrial network associated with PGC-1α silencing characterized by loss of tubular organization and formation of donut/blob-shaped mitochondria, a hallmark of mitochondrial dysfunction (Ahmad et al, 2013) (Fig 1C). Such transition from tubular to globular shape was correlated with the transcriptional repression of the mitochondrial fission/fusion dynamics genes FIS1 and MFN2 (Fig 1D). Change in mitochondrial mass was evaluated by quantifying the activity of citrate synthase, the enzyme catalyzing the first reaction of the Krebs cycle, and a superior biomarker of mitochondrial content (Steen et al, 2012). Despite the well-established function of PGC-1α in promoting mitochondrial biogenesis, citrate synthase activity (CSA) was found to be significantly higher in shPGC1A cells at all time-points studied (Fig 1E), whereas the expression of the mitochondrial replication-related genes *POLG* and *TFAM* remained unchanged except for a small induction of *TFAM* after 21 d (Fig S1B). This unexpected increase in CSA activity is likely reflecting an adaptive response to metabolic/oxidative stress as previously shown in pancreatic cancer (Schlichtholz et al, 2005; Vishnyakova et al, 2016). Indeed, evaluation of mitochondrial OXPHOS by extracellular flux analysis and measurement of the oxygen consumption rate (OCR) revealed that loss of PGC-1⍺ decreased all phases of respiration as early as day 7 (Fig 1F). Conversely, maximal and reserved glycolytic capacities of shPGC-1α cells were significantly increased (Fig S1C and D). Although shPGC-1α cells display enhanced glycolytic capacity, cellular ATP content was lower at all time points (Fig S1E). These changes were accompanied by increased mitochondrial superoxide production after day 14 (Fig 1G), whereas general ROS content was higher at all time points (Fig 1H). ROS accumulation in PGC-1α–silenced cells was associated with repression of catalase (*CAT*) expression at days 7, 14, and 21 (Fig 1I), whereas cytoplasmic superoxide dismutase (*SOD1*), mitochondrial thioredoxin (*TXN2*), and glutathione peroxidase (*GPX*) genes were found to be significantly down-regulated at day 21 (Fig 1I). Repression of antioxidant enzymes was also associated with down-regulation of the mitochondrial sirtuin (sirtuin 3, *SIRT3*), a known target of PGC-1α transcriptional induction with important roles in ROS suppression and mitochondrial function (Kong et al, 2010) (Fig 1J). Surprisingly, sirtuin-1, a key upstream regulator of PGC-1α activity (Cantó & Auwerx, 2009), was also found to be repressed after sustained PGC-1α loss of function (Fig 1J). To determine if some of the changes observed could be attributed to potential compensatory induction of PGC-1 isoforms, gene expression of PGC-1β and PRC were quantified and found to not be statistically different between shPGC1A and shCtrl cells (Fig 1K). Together, these findings indicate that PGC-1α is a critical regulator of RPE mitochondrial dynamics, function, and cellular redox state.

### PGC-1α silencing impairs RPE autophagic flux

**Figure 2.**
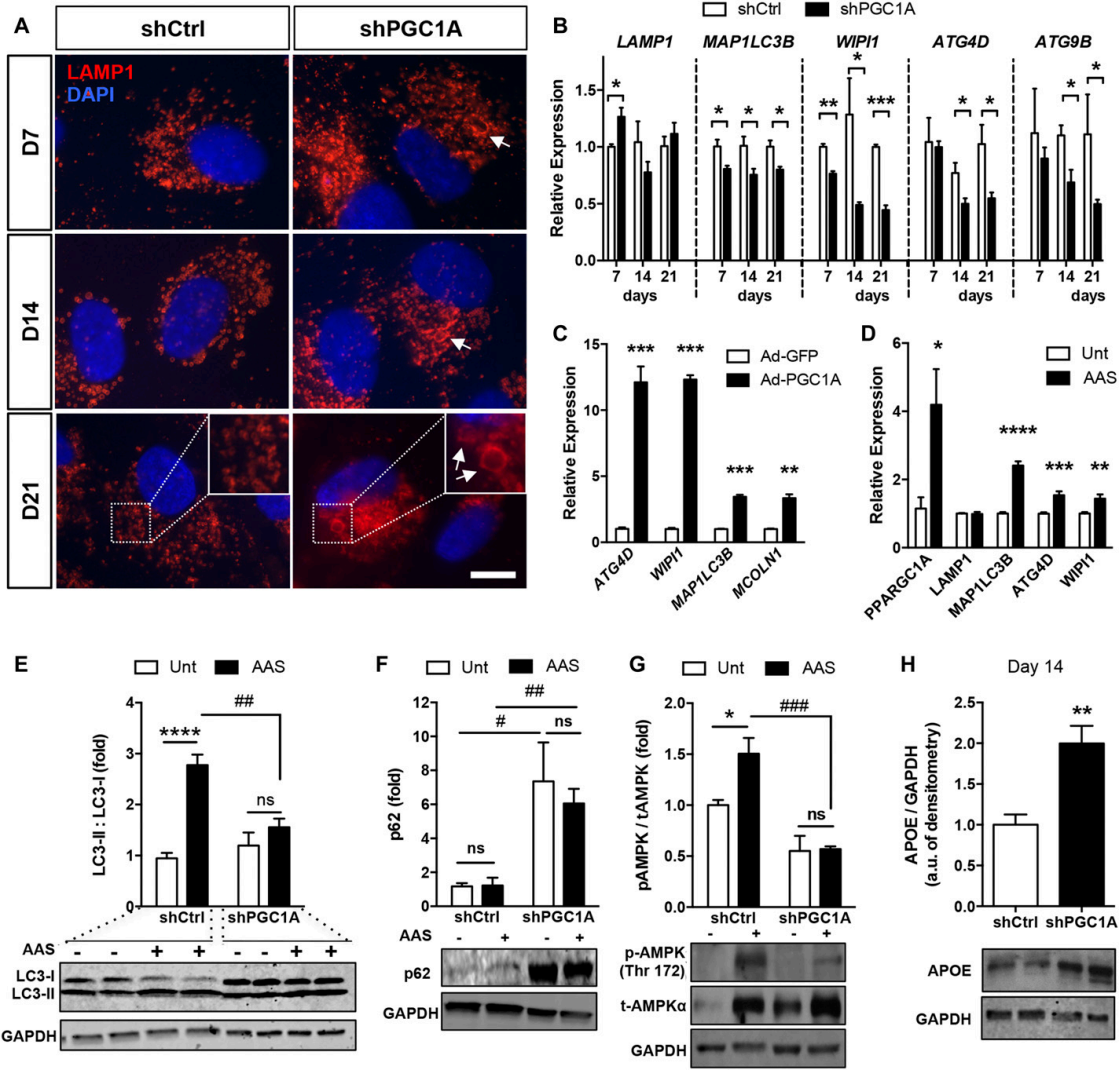
PGC-1α activation is required for autophagy flux initiation in ARPE-19 cells. **(A)** Immunodetection of late-stage endosomes (LAMP-1, red) in shPGC1A and shCtrl ARPE-19 matured for up to 21 d. Arrows indicate dilated late endosomes in shPGC1A cells. **(B)** Expression profiles of autophagy-related genes MAP1LC3B and WIPI (initiation of autophagosomes), ATG4D (autophagosome maturation), and ATG9B (lysosomal degradation pathway) (n = 3–4, per condition and time points). Scale bar is 10 μm. **(C)** Changes in autophagosome biogenesis-related genes ATG4D, WIPI1, MAP1LC3B, and MCOLN1 in parental ARPE-19 cells 24 h after adenoviral-mediated PGC-1α overexpression (n = 3). **(D)** PGC-1α and autophagy-related genes LAMP1, MAP1LC3B, ATG4D, and WIPI1 expression quantified by qPCR in parental ARPE-19 cells after AAS for 24 h. Untreated (Unt) cells were used as controls (n = 4). **(E–G)** Western blot analysis for LC3B (E); polyubiquitin-binding p62 protein sequestosome (p62) (F); phospho (Thr172) and total AMPKα (G) in shCtrl and shPGC1A cells 2 h after AAS. Immunoblots are representative of n = 3–4 experiments per conditions. **(H)** APOE protein expression in shCtrl and shPGC1A cells ARPE-19 cells after 14 d of maturation (n = 4). For all blots, GAPDH was used as the loading control. Error bars are means ± SEM. Data were analyzed by multiple unpaired *t* test comparisons using the Holm-Sidak method (B–D, H) or ANOVA followed by Tukey’s multiple comparison test (E–G). *P ≤ 0.05; **P ≤ 0.01; ***P ≤ 0.001; ****P ≤ 0.0001 versus respective controls. ^#^*P* ≤ 0.05, ^##^*P* ≤ 0.01 and ^###^*P* ≤ 0.001 = shPGC1A versus shCtrl under AAS.

**Figure S2.**
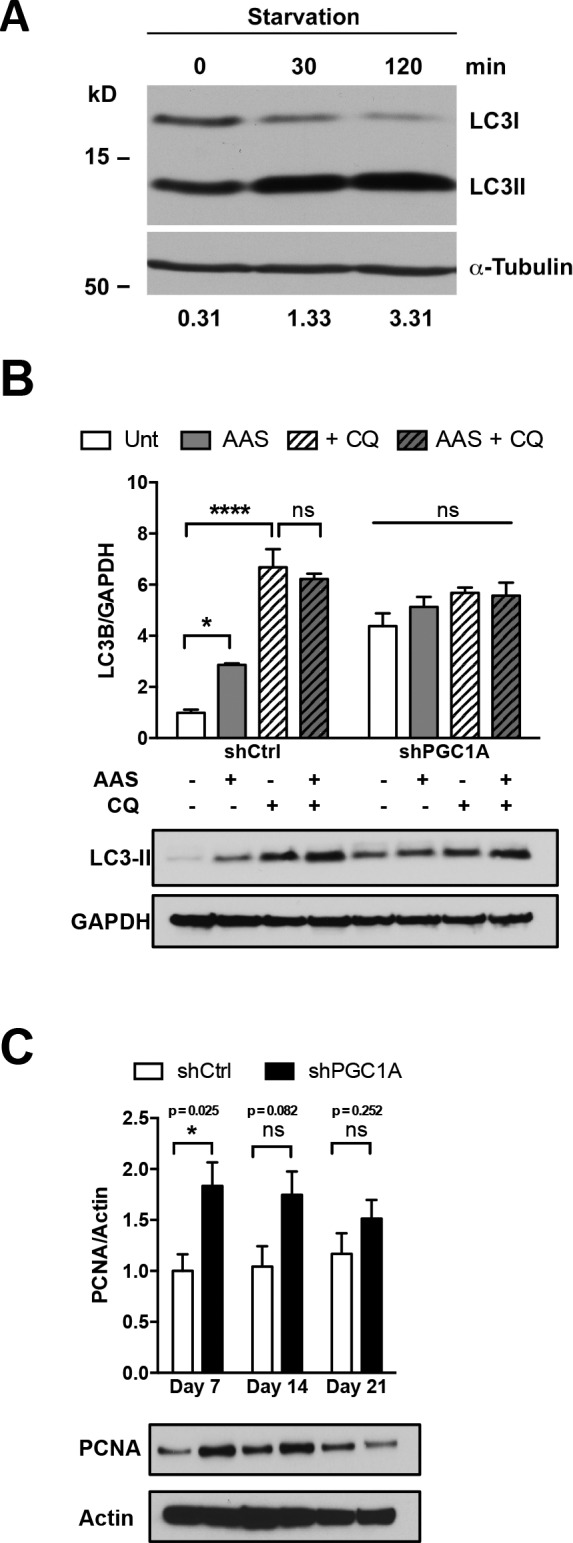
AAS-dependent induction of autophagic flux in ARPE-19. **(A)** Quantification of LC3I to LC3II protein conversion after AAS for 30 and 120 min in parental ARPE-19. Densitometric analysis of LC3II/LC3I ratios shows robust conversion (≈10-folds) at 2 h of treatment. **(B)** Densitometric analysis of LC3-II protein levels relative to GAPDH by Western blotting after AAS and/or CQ treatment in shCtrl and shPGC1A cells. Treatment of shPGC1A cells with CQ shows no increased accumulation of LC3II compared with shCtrl. Error bars represent mean ± SEM. Two-way ANOVA with Tukey’s post hoc test (n = 4). ***P ≤ 0.001; ****P ≤ 0.0001. **(C)** Densitometric analysis of PCNA protein levels relative to Actin by Western blotting in shCtrl and shPGC-1α ARPE-19 cells collected at 7, 14, and 21 d (n = 4). Error bars represent mean ± SEM. Data were analyzed by multiple unpaired t-test comparisons using the Holm–Sidak method. *P ≤ 0.05 compared with their respective shCtrl at each time point.

RPE homeostasis and functions are tightly dependent on regulated autophagic activity (Kaarniranta et al, 2013) and failure to degrade damaged cellular organelles and molecules under pro-oxidative conditions can trigger RPE dysfunction (Saadat et al, 2014; Zhang et al, 2015; Golestaneh et al, 2017). To evaluate the effect of PGC-1α silencing on the lysosomal/autophagic status of RPE cells, we first examined the organization of late-endosomes stained with lysosomal-associated membrane protein 1 (LAMP-1) during RPE maturation and observed at all time points the presence of highly dilated LAMP-1+ lysosomes in shPGC1A cells compared with control cells, where endosomes maintained a uniformly small size (Fig 2A). Based on these observations, we examined whether expression of autophagy-associated genes could be affected in shPGC1A cells and found that MAP1LC3B and WIPI, both involved in the initiation and lengthening of the autophagosomes, were suppressed from day 7, whereas ATG4D, involved in late stages of autophagosome maturation and ATG9B, which modulates the lysosomal degradation pathway, was reduced at 14 and 21 d compared with control conditions (Fig 2B). Contrariwise, adenovirus-mediated PGC-1α overexpression strongly induced the expression of autophagy-related genes in parental ARPE-19 (Fig 2C). To provide further evidence of a direct role for PGC-1α in regulating RPE autophagic activity, parental ARPE-19 cells were subjected to amino acid starvation (AAS) for 24 h to promote autophagic flux (Martina et al, 2016) and PGC-1α was found to be significantly induced along with other genes implicated in autophagy and lysosomal biogenesis (Fig 2D). We next evaluated the consequences of PGC-1α loss-of-function on RPE autophagic flux, by monitoring the conversion of LC3 from the soluble cytoplasmic form LC3-I to the lipid-bound form LC3-II (Jiang et al, 2015) after 2 h of AAS, the optimal time point for LC3-I to LC3-II conversion in parental ARPE-19 cells (Fig S2A). Whereas the expected increase in LC3-II/LC3-I ratio was observed in starved shCtrl cells, no LC-3 conversion was detected in shPGC1A cells (Fig 2E), suggesting that in the absence of PGC-1α, the cells were no longer able to induce autophagy to maintain an homeostatic amino acid content. Assessment of the autophagic flux by use of the lysosomal inhibitor chloroquine (CQ) revealed that, contrary to shCtrl cells, LC3-II level in shPGC1A cells was not increased by CQ (Fig S2B), confirming that silencing PGC-1α impairs RPE autophagic flux. The defective autophagic response in shPGC1A cells was further demonstrated by accumulation under both basal and AAS conditions of the ubiquitin-binding adaptor proteins such as p62/SQSTM1 that binds LC3 and is, therefore, degraded by autophagy (Fig 2F). Activation of AMPK, the major regulator of autophagy initiation, measured as the ratio of phospho-Th172 to total AMPKα, was increased in shCtrl cells exposed to AAS but not in shPGC1A cells (Fig 2G), showing that PGC-1α is critical for amino acid–dependent AMPK activation. As susceptibility to oxidative stress and impaired autophagy was observed to be accompanied by the up-regulation of AMD-associated genes such as apolipoprotein E (APOE) expression in primary RPE cells from AMD patients (Golestaneh et al, 2017), we quantified the expression of APOE and found its protein level increased in shPGC-1α RPE cells at 14 d (Fig 2H).

### Sustained PGC-1α silencing induces RPE dedifferentiation and mesenchymal transition

**Figure 3.**
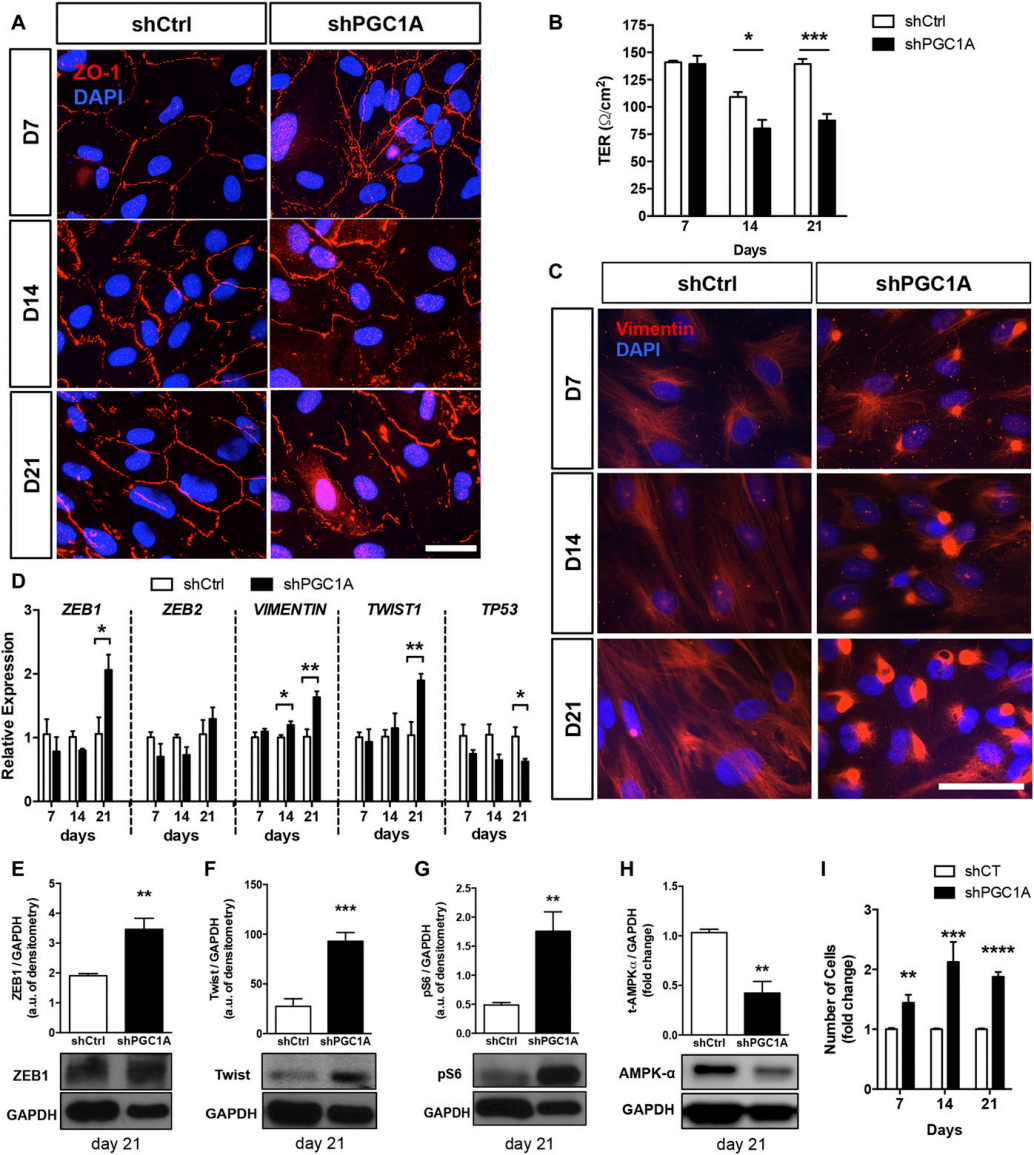
Sustained PGC-1α loss of function triggers mesenchymal transition. **(A)** Immunodetection of the tight junction protein ZO-1 (red) in shPGC1A and shCtrl ARPE-19 cells matured for up to 21 d. Scale bar is 10 μm. **(B)** Barrier integrity measured by TER (n = 3 per condition). **(C)** Immunostaining of the vimentin IFs (red) in shPGC1A and shCtrl ARPE-19 cells matured from 7 up to 21 d. DAPI (blue) was used to stain nuclei. Scale bar is 50 μm. **(D)** Expression of EMT-related genes ZEB1, ZEB2, VIM, TWIST, and the cell cycle regulator p53 measured by qPCR analysis (n = 3–4). **(E–H)** Protein levels of the EMT transcriptional factors ZEB1 (E) and TWIST1 (F), the mTOR-dependent phospho-S6 ribosomal protein (Ser235/236) (G) and total AMPKα (H) in shPGC1A and shCtrl cells at 21 d. Immunoblots are representative of n = 3–4 samples per conditions. **(I)** Quantification of cell number per well expressed as fold change to shCtrl for each time point demonstrating significant increases in cell density in shPGC1A cells (n = 8–15). Error bars are means ± SEM. Data were analyzed by unpaired *t* test. *P ≤ 0.05; **P ≤ 0.01; ***P ≤ 0.001; ****P ≤ 0.0001 compared with their respective shCtrl at each time points.

To investigate the phenotypic consequences of the mitochondrial and autophagic dysfunction triggered by PGC-1α silencing, integrity of the RPE barrier was assessed by immunostaining of the tight junction protein ZO-1, normally localized at the cell membrane of mature RPE. When compared with shCtrl cells, ZO-1 was found to be disorganized and limited to no membranous localization at days 14 and 21 in shPGC1A (Fig 3A). Failure of shPGC1A RPE cells to form and maintain proper tight junctions was confirmed by measuring transepithelial electrical resistance (TER) of the cells (Fig 3B). As progressive loss of barrier function may be caused by epithelial dedifferentiation, RPE monolayers were stained for vimentin whose induction is associated with RPE mesenchymal transition (Adijanto et al, 2012) and oxidative/nitrosative stress (Sripathi et al, 2012). Whereas the vimentin intermediate filaments (IFs) formed an evenly distributed arborized network in shCtrl cells, gradual disorganization and condensation with prominent perinuclear bundling of the IFs was observed in shPGC1A cells (Fig 3C). We then asked whether the observed collapse and entanglement of vimentin IFs could be indicative of EMT. Gene expression analysis showed induction of vimentin expression at days 14 and 21 and of the EMT transcription factors *ZEB1* and *TWIST1* at day 21 concomitant with repression of *P53* (Fig 3D). Protein expression analysis in cells matured for 21 d confirmed the significant induction of ZEB1 and Twist (Fig 3E and F) along with mTOR-dependent pS6 ribosomal protein (Ser93) activation (Fig 3G) and decreased total levels of AMPK-α (Fig 3H) in shPGC1A cells. Importantly, this phenomenon was associated with unregulated cell division as indicated by increased cell density in PGC-1α–silenced cells compared with nonsilenced controls (Fig 3I). Moreover, assessment of cellular proliferation by quantification of proliferating cell nuclear antigen (PCNA) levels indicated higher proliferation in shPGC-1α cells at day 7 but not days 14 and 21 (Fig S2C). Taken together, these data indicate that PGC-1α is required to maintain RPE epithelial phenotype and that sustained PGC-1α loss triggers EMT in RPE.

### PGC-1 deletion in adult mice causes RPE dysfunction and neurodegeneration

**Figure S3.**
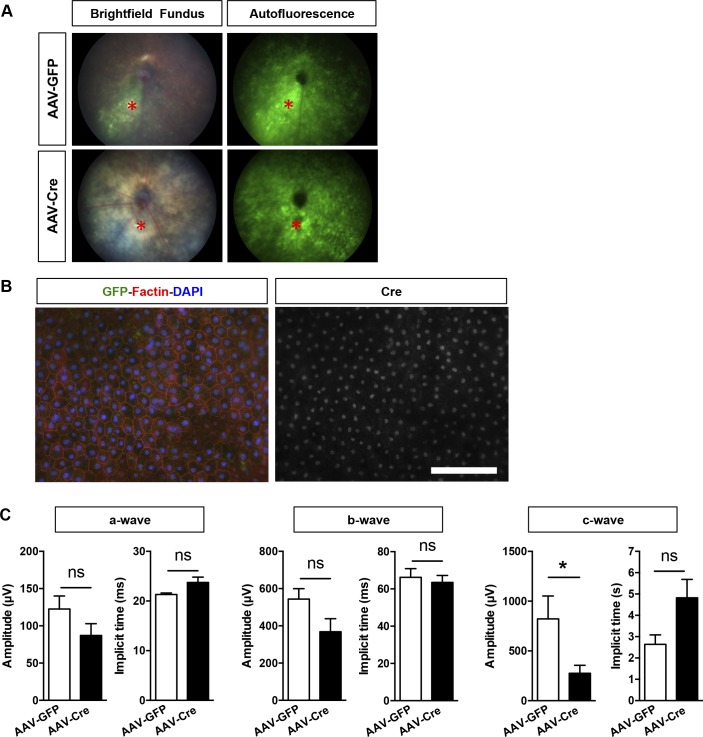
Morphological and functional evaluation of adult PGC-1–floxed mice 2 and 4 wk after subretinal AAV injection. **(A)** Example of fundus imaging GFP fluorescence detection confirming the pan-retinal transduction of the AAVs 2 wk after subretinal injection in experimental floxed animals. The asterisk (*, red) indicates the site of injection. **(B)** RPE/choroid flat mounts immunostained for F-actin (red) and Cre (white) from AAV-Cre–injected experimental mice revealing Cre+ nuclei in a large area of the epithelium 1 mo postinjection. No major morphological changes were observed. Scale bar is 100 μm. **(C)** ERG analysis of the *a*-, *b*-, and *c*-wave amplitudes and implicit times 1 mo postinjection (n = 5–6). Error bars are means ± SEM. Unpaired *t* tests were used in all comparisons. *P ≤ 0.05 compared with their respective AAV-GFP mice.

**Figure 4.**
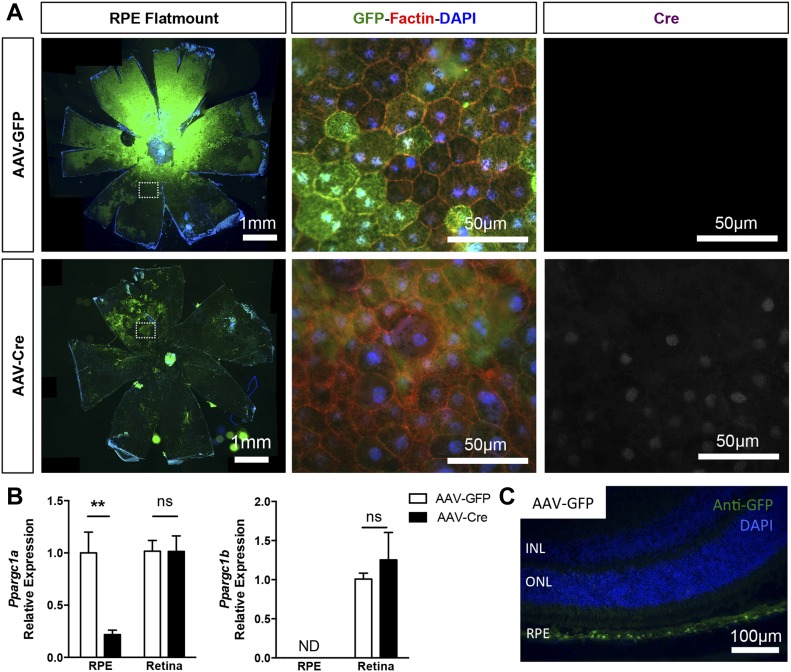
RPE-specific deletion of PGC-1 isoforms in adult mice. **(A)** RPE/choroid flat-mounted preparations from adult PGC-1α;PGC-1β–double floxed mice 1 mo after subretinal injection with AAV-GFP and AAV-Cre. Immunolocalization of the GFP reporter (green) confirmed efficient viral transduction of the entire RPE surface. High magnifications in the mid-periphery (dotted squares) show GFP+ RPE cells labeled with F-actin (red). Cre expression (white) was only detected in the nuclei of RPE from AAV-Cre–injected animals. **(B)** PGC-1α and PGC-1β gene expression analysis in RPE/choroid and retinal tissues from AAV-GFP and AAV-Cre mice 1 mo postinjection (n = 3–6). **(C)** Representative ocular cryosection from experimental mice 4 mo after AAV-GFP injection, showing highly selective viral transduction of the RPE cells. Scale bar is 100 μm. Error bars are means ± SEM. Data were analyzed by unpaired *t* test. **P ≤ 0.01 compared with their respective AAV-GFP control mice.

To validate our findings in vivo, we developed and characterized a mouse model of conditional PGC-1α deletion in adult RPE by AAV-Cre recombination of PGC-1–floxed animals. To bypass any potential compensatory responses from PGC-1β (Sczelecki et al, 2014), which is normally expressed at low to undetectable levels and inversely correlated to PGC-1α levels in matured RPE (Iacovelli et al, 2016), we elected to ablate both isoforms using double PGC-1α/β–floxed animals (Rowe et al, 2013). The RPE specificity of our transgene expression was achieved by subretinal delivery and titration of the RPE trophic AAV2/8 (Vandenberghe et al, 2011). At 2- and 4-wk post-AAV injection in experimental floxed animals, we used fundus photography (Fig S3A) and RPE/choroid flat mounts to confirm the efficient transduction by AAV2/8-CASI-GFP (AAV-GFP) and AAV2/8-CASI-Cre (AAV-Cre) of most of the RPE area (Fig 4A). Co-staining for GFP, F-actin, and Cre on RPE flat mounts showed that 1-mo after AAV-GFP or AAV-Cre injection, the RPE monolayer maintained a normal cuboidal morphology and that Cre expression was only detected in the nuclei of RPE from AAV-Cre–injected animals (Figs 4A and S3B). Gene expression analysis performed on isolated RPE and neuroretinal tissues showed reduction by 80% of PGC-1α mRNA in the RPE of AAV-Cre–injected eyes compared with AAV-GFP controls, whereas PGC-1β was undetectable in both conditions and unchanged in the retina (Fig 4B). Immunodetection of GFP+ cells on ocular cryosections from AAV-GFP–injected mice confirmed the RPE trophism of the virus under our conditions (Fig 4C).

**Figure 5.**
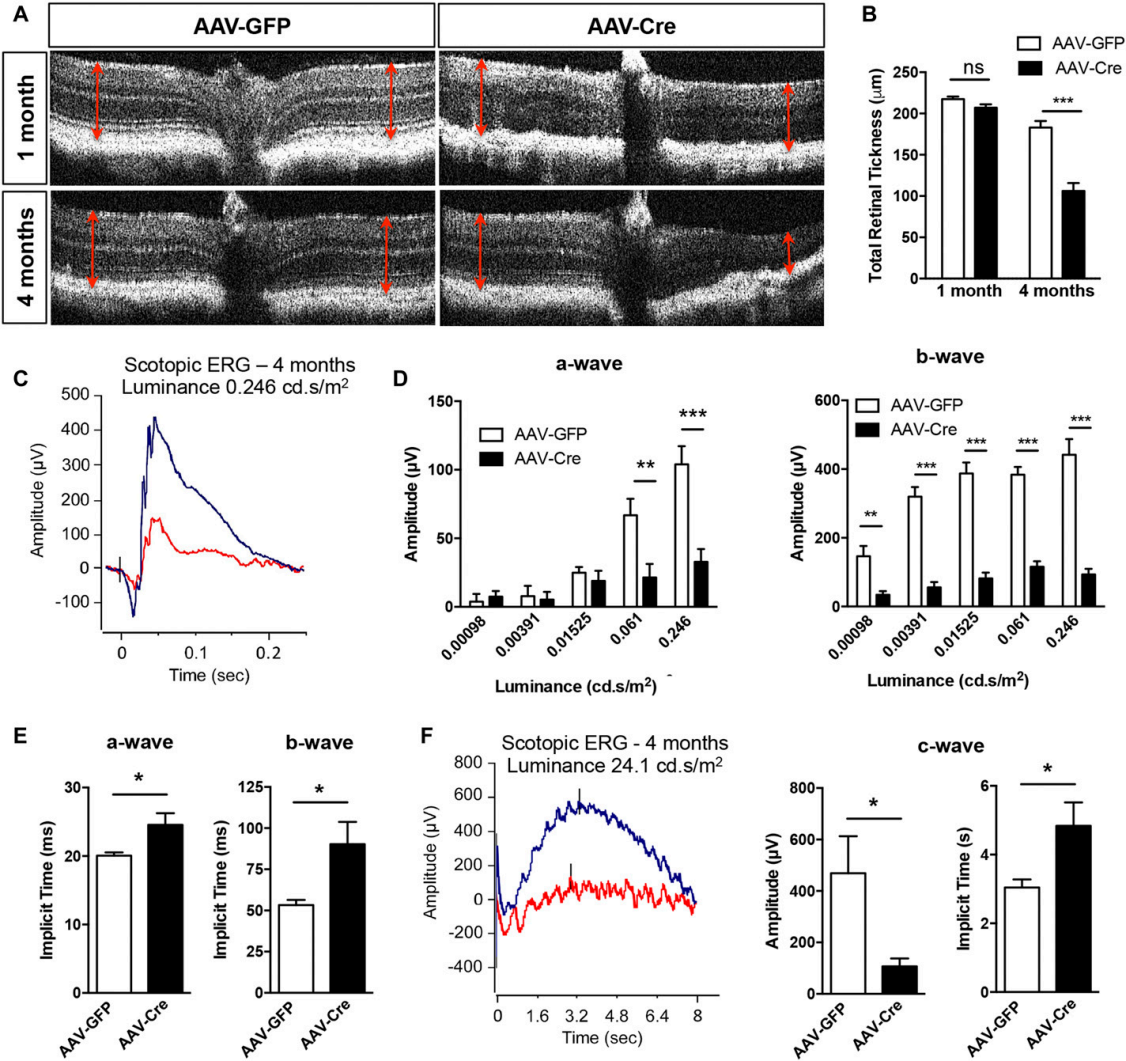
Morphologic and functional evaluation of adult conditional PGC-1 knockout mice. **(A)** Representative OCT images from AAV-GFP and AAV-Cre–injected PGC-1A;B–floxed mice 1 and 4 mo postinduction. **(B)** Quantification of total retinal thickness from OCT imaging (n = 5–7). **(C)** Scotopic ERG recordings at a luminance of 0.246 (P) cd.s/m^2^ from AAV-GFP (blue) and AAV-Cre (red) animals. **(D)** Quantification of *a*- and *b*-wave amplitudes for increasing stimulus intensity at 4 mo (n = 5). **(E)** Implicit times of *a*- and *b*-wave at light stimulus of 0.246 (P) cd.s/m^2^ at 4 mo (n = 5). **(F)** RPE-generated *c*-wave amplitude and implicit time in AAV GFP group mice (blue) compared with AAV-GFP injected mice (red) measured with a light stimulus of 24.1 cd.s/m^2^ at 4 mo (n = 6). Error bars are means ± SEM. Data were analyzed by unpaired *t* test. *P ≤ 0.05; **P ≤ 0.01; ***P ≤ 0.001 compared with their respective AAV-GFP controls.

**Figure S4.**
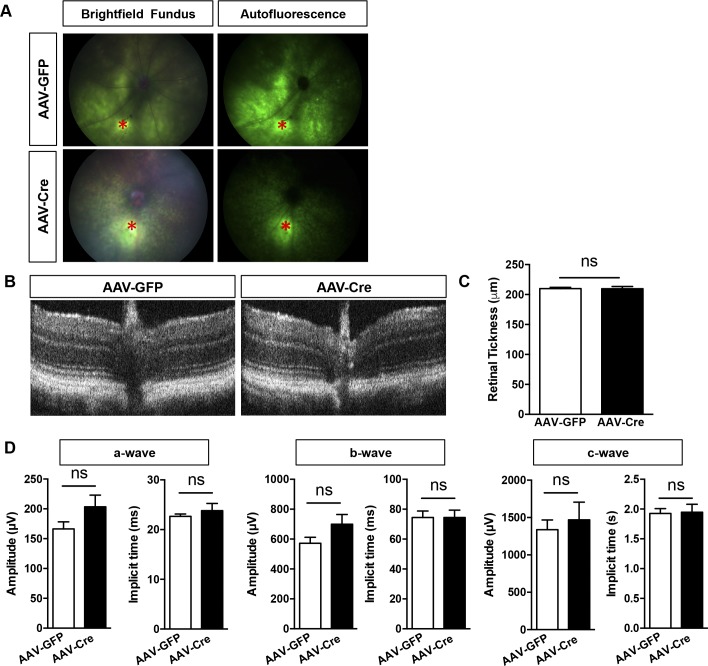
OCT and ERG analysis of wild-type mice 2 and 4 wk after injection with AAVs. **(A)** Fundus photography and direct GFP fluorescence detection 2 wk postinjection of the AAVs in WT C57Bl/6J animals. The site of injection is marked by an asterisk (*, red). **(B)** Representative OCT scans indicating normal morphology and lamination 1 mo postinjection with AAV-GFP and AAV-Cre. **(C)** Quantification of total retinal thickness indicating no change in GFP- versus Cre-transduced WT animals (n = 5). **(D)** Scotopic ERG measurements of the *a*-, *b*-, and *c*-wave amplitudes and implicit time 1 mo postinjection (n = 12). Error bars are means ± SEM. Unpaired *t* tests were used in all comparisons. No statistical difference was determined between the cohorts.

Monitoring of retinal morphology by optical coherence tomography (OCT) and retinal thickness measurement did not reveal any significant changes in AAV-Cre mice 1 mo postinjection besides some loss of retinal lamination (Fig 5A and B), but scotopic electroretinogram (ERG) recording showed a trend, although nonstatistically significant, toward a reduction of the *a*- and *b*-wave amplitudes, whereas the RPE generated *c*-wave was decreased by ≈66% (Fig S3C). At 4 mo, a significant decrease in total retinal thickness measured by OCT was observed in AAV-Cre–injected PGC-1–floxed mice compared with AAV-GFP group (Fig 5A and B). Functional evaluation of the retina and RPE by ERG revealed severe reduction of the *a*-, *b*-, and *c*-wave amplitudes and delayed peak time of response (Fig 5C–F). As a control, OCT, and ERG analysis were also conducted in wild-type C57BL/6J injected with AAV-GFP and AAV-Cre and showed no alteration in morphology (Fig S4A–C) and function (Fig S4D).

### PGC1 deletion drives RPE disorganization and loss of epithelial integrity

**Figure 6.**
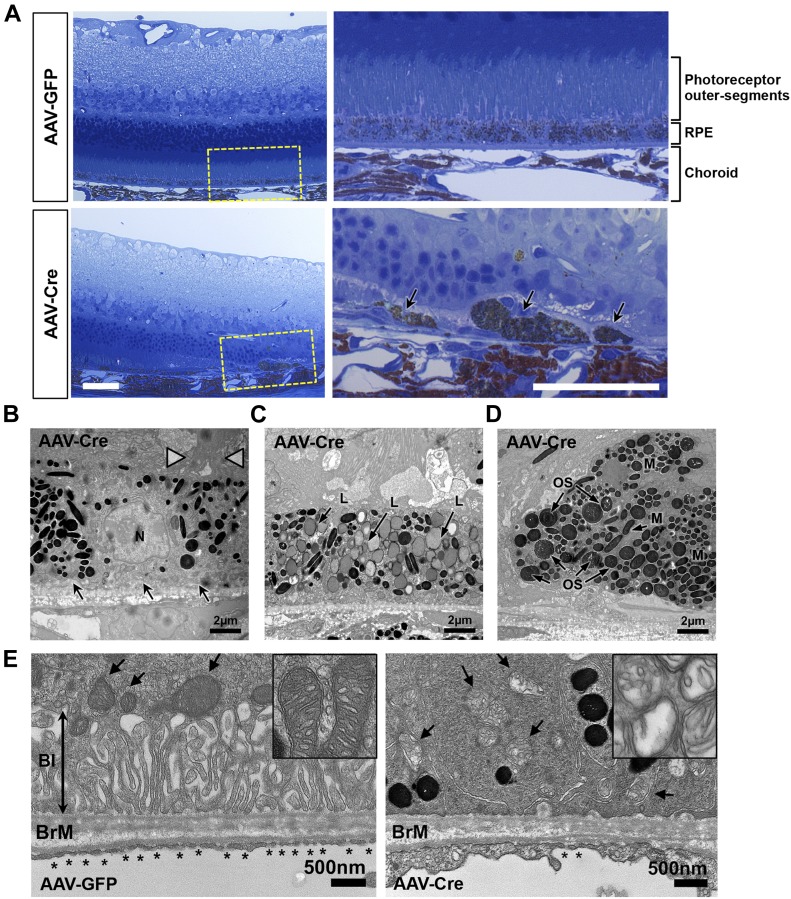
PGC-1 deficiency triggers gross morphological changes in the outer retina and RPE. **(A)** Light micrographs from toluidine blue–stained semithin ocular sections from AAV-GFP and AAV-Cre–injected double floxed mice 4 mo postinjection showing severe disorganization of the RPE layer associated with prominent neuroretinal degeneration. Higher magnification of the RPE/choroid complex (corresponding to the dashed yellow boxes) shows degenerative lesion with swollen and sloughed RPE cells in the AAV-Cre–injected mice (arrows). Scale bars are 50 μm. **(B–D)** Transmission electron micrographs showing three representative examples of abnormal RPE in AAV-Cre–injected mice. Observed phenotypic changes vary from disorganized apical microvilli (arrowheads) with loss of basal infoldings (arrows) (B), massive accumulation of lipofuscin-containing granules (L) (C) to enlarged and dissociated cells filled with melanosomes (M) and undigested outer segments (OS) (D). Scale bars are 2 μm. **(E)** High magnification of the RPE basal side highlighting the prominent loss of basal infoldings (BI, double arrow), loss of fenestrations (*), thickening of the choriocapillaris endothelium, and severe mitochondria degeneration (arrows) marked by ruptured outer mitochondrial membranes and cristae remodeling (see insert). Scale bars are 500 nm.

Histological evaluation of the ocular posterior segments of experimental animals 4 mo postinjection revealed regional RPE dysmorphia in AAV-Cre mice with enlarged and highly pigmented cells detached from the epithelial monolayer and surrounded by thick extracellular matrix deposition (Fig 6A). These regions of RPE degeneration were associated with marked photoreceptor thinning and loss of outer segments (OS) (Fig 6A). Ultrastructural analysis of the OS/RPE/choriocapillaris interface by TEM imaging revealed various stages of RPE phenotypic anomalies, characterized by severe loss of both apical microvilli and basal infoldings (Fig 6B and C); accumulation of large lipofuscin-filled granules (Fig 6C), melanosomes, and phagosomes with undigested OS (Fig 6D); thickening of the choriocapillaris endothelium and loss of fenestrations (Fig 6E); severe mitochondrial degeneration with swelling and loss of the cristae and incomplete outer-membrane closure characteristic of “mitochondria membrane ghosts” (Fig 6E).

### PGC-1 deletion promotes RPE EMT in vivo

**Figure 7.**
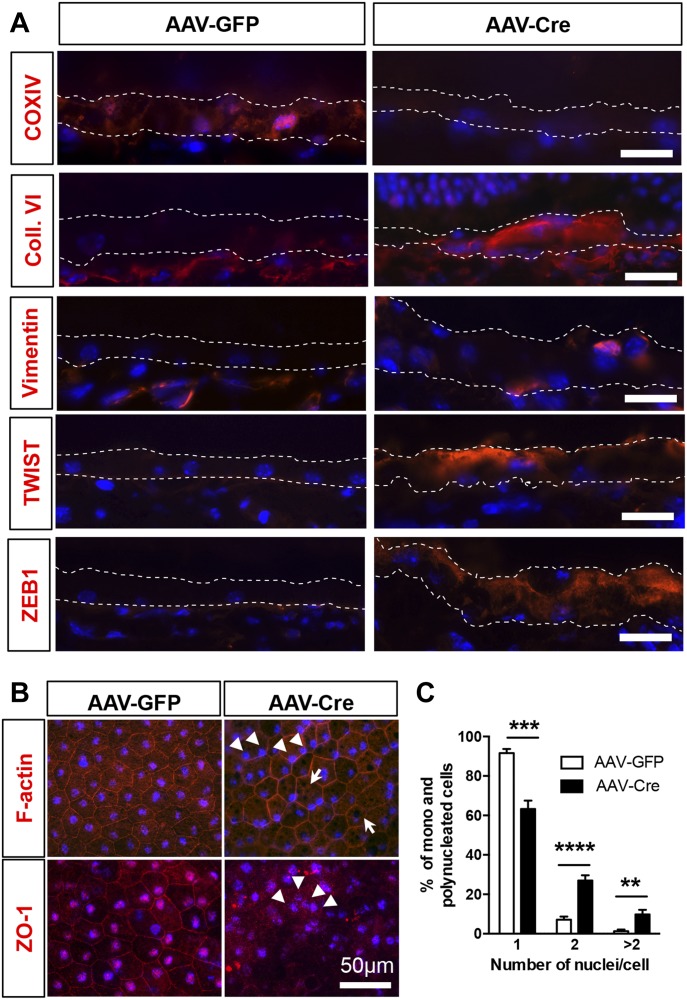
RPE dedifferentiation and mesenchymal transition after PGC-1 deletion. **(A)** Immunodetection of mitochondrial and EMT markers on cryosections of eyes collected 4 mo postinjection with AAV-GFP or AAV-Cre, showing marked loss of the ECT component COXIV in the RPE layer (defined by the white dotted lines) of the AAV-Cre–injected animals, whereas the pro-EMT transcription factors TWIST1 and ZEB1 were strongly up-regulated. Mesenchymal phenotypic transition is further demonstrated by increased collagen VI deposition and vimentin perinuclear condensation. Scale bars are 20 μm. **(B)** RPE flat-mount preparation immunostained for F-actin or ZO-1 (red), demonstrating increased multinucleated cells (arrowheads) with large pigmented vacuoles (arrows) and loss of membranous ZO-1 distribution. Nuclei were costained with DAPI (Blue). Scale bar is 50 μm. **(C)** Percentage of mononucleated, binucleated, and polynucleated (>2 nuclei/cell) cells quantified on F-actin stained RPE flat mounts (n = 6). Error bars are means ± SEM. Data were analyzed by unpaired *t* test. **P ≤ 0.01; ***P ≤ 0.001; ****P ≤ 0.0001 compared with AAV-GFP group.

**Figure S5.**
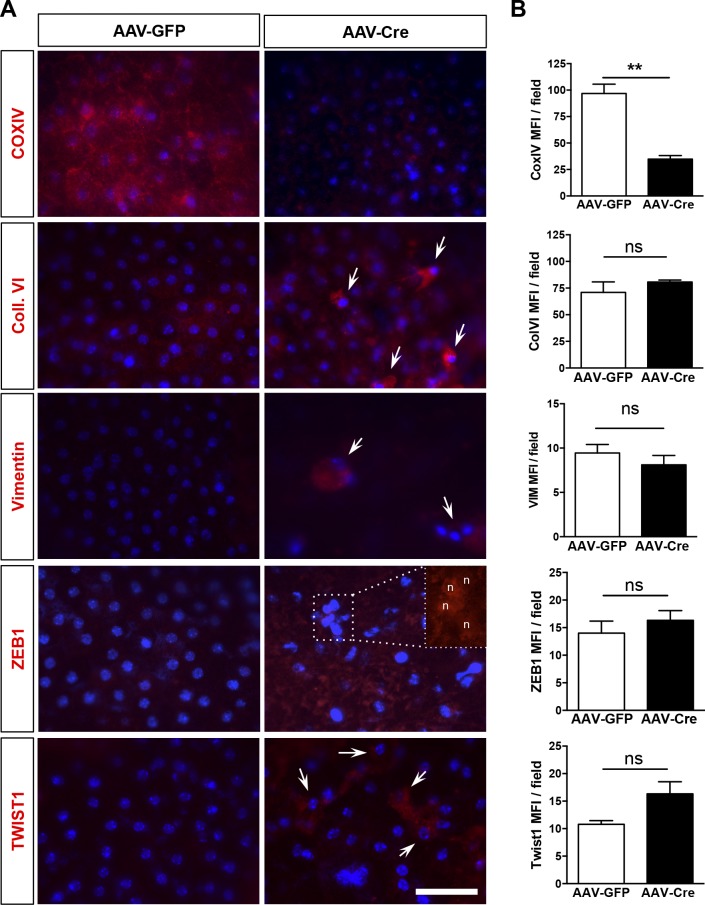
Immunolocalization and quantification of mitochondrial and EMT markers in PGC-1–floxed mice 4 mo after subretinal AAV injection. **(A)** Representative images of RPE flat-mount preparations from AAV-GFP and AAV-Cre–injected animal at 4 mo immunostained for the mitochondrial marker, COXIV, and the EMT markers, Coll. VI, Vimentin, ZEB1, and TWIST1 (red) and costained with DAPI (blue). Whereas depletion of COXIV was observed in all fields of AAV-Cre–injected mice, increases in the EMT markers were only observed in localized areas with abnormal and sloughed RPE (arrows). Insert in the ZEB1 panel shows nuclear localization of the EMT transcription factor in amorphic RPE cells (n = nucleus). Scale bar is 50 μm. **(B)** Fluorescence intensity quantification indicated a significant reduction of COXIV mean fluorescence intensity by 60% after AAV-Cre. Mean fluorescence intensity of the EMT-markers was not significantly altered as the changes were localized (4–6 randomly selected fields per sample, n = 3). Error bars are means ± SEM. Unpaired *t* tests were used in all comparisons. **P ≤ 0.01 compared with their respective AAV-GFP group.

Based on the morphological evidence of RPE dedifferentiation and our in vitro findings, we evaluated markers for oxidative phosphorylation, mesenchymal transition, and epithelial integrity by immunofluorescence on cryosections. Evaluation of regions associated with overt retinal degeneration and RPE phenotypic changes showed decreased detection of the mitochondrial marker COX-IV after PGC-1 deletion, whereas the EMT markers—collagen-VI, vimentin, TWIST1, and ZEB1- were increased (Fig 7A). Interestingly, vimentin was also found bundled perinuclearly as in our in vitro model of PGC-1α silencing. Evaluation of global alteration in mitochondrial and EMT markers by staining and quantification of RPE/choroid flat mounts showed that whereas COX-IV was uniformly repressed in the RPE, EMT markers were only found increased in a subset of amorphic RPE cells (Fig S5). Investigation of the cytoskeletal and tight junction organization by phalloidin/DAPI staining on RPE/choroid flat mounts revealed in AAV-Cre mice a loss of hexagonal shape, increased multinucleation, and accumulation of large pigmented vacuoles (Fig 7B and C), a phenotype associated with age-related oxidative stress (Chen et al, 2016). Similar to our in vitro study, ZO-1 was found disorganized and absent in some regions (Fig 7B). Taken together, our results show that PGC-1α expression is required for the maintenance of RPE epithelial phenotype and function. The RPE dysfunction and mesenchymal transition caused by PGC-1α deletion is associated with disorganization of the outer retinal complex and severe retinal degeneration.

## Discussion

Our results show that PGC-1α is critical for the maintenance of RPE homeostatic processes, including glucose metabolism, autophagy, and epithelial integrity. Our longitudinal analysis in vitro indicates that PGC-1α loss primarily alters RPE metabolic and autophagic functions followed by EMT induction. In vivo deletion of PGC-1α in adult RPE triggers loss of the epithelial phenotype and cellular disorganization associated with choroidal and neuronal degeneration. The phenotypic changes we observed in our mouse model of PGC-1α deletion in adult RPE are remarkably similar to that described recently in aged NRF2/PGC-1α double knockout animals showing impaired autophagy, increased oxidative damage, and damaged mitochondria in RPE (Felszeghy et al, 2019). Interestingly, global NRF2/PGC-1α deficiency was also characterized by increased P62 and LC3B combined with accumulation of melanosomes, autolysosomes, and abnormal mitochondria (Felszeghy et al, 2019).

In postmitotic cells, autophagy is the major mechanism for renewing damaged organelles and recycling nutrients. Mitochondrial health, antioxidant capacity, and autophagy are thereby tightly interconnected processes regulated by complex positive and negative feedback loops. Mitochondrial oxidative damage and/or decreased ATP levels can directly impair lysosomal activity (Demers-Lamarche et al, 2016), and defective lysosomal degradation can reciprocally cause the accumulation of defective mitochondria (Osellame et al, 2013). As PGC-1α transcriptionally induces numerous genes involved in all of these processes, teasing out the initial pathway(s) affected by PGC-1α silencing in RPE is difficult, but our longitudinal analysis offers interesting mechanistic cues. Our group has previously shown that PGC-1α is strongly induced during RPE functional maturation and that PGC-1α gain of function promotes (i) RPE metabolic function through induction of mitochondrial complex proteins and (ii) resistance to cytotoxic stress by inducing essential mitochondrial and cytoplasmic antioxidant enzymes (Iacovelli et al, 2016; Satish et al, 2018). Here, we show that, conversely, PGC-1α deficiency in RPE led to rapid loss of mitochondrial OXPHOS and reduced expression of catalase. These two early events may be key in promoting the increase in ROS generation and autophagic dysfunction, which ultimately caused the loss of epithelial status in RPE. The rise in total ROS appeared to precede the detection of higher mitochondrial superoxide production which could be explained by an increase in mitochondrial calcium influx after mitochondrial stress (Ahmad et al, 2013) or reflect enhanced mitochondrial production of hydrogen peroxide and hydroxyl radical (Kirkland & Franklin, 2001).

PGC-1α is well known as a major transcriptional inducer of mitochondrial biogenesis. However, PGC-1α silencing in RPE did not alter the expression of the mitochondrial replication-related genes *TFAM* and *POLG* expression nor grossly reduce mitochondrial content. Even more unexpected was the detection of a robust increase in CS activity in shPGC-1α cells. Although CS activity is a well-accepted biomarker of mitochondrial mass and oxidative capacity, our findings suggest that CS activity may not be directly correlated with mitochondrial content in metabolically defective RPE cells. Indeed, mature and healthy RPE cells have been shown to preferentially use reductive carboxylation of α-ketoglutarate to produce citrate (Du et al, 2016) and induction of CS may, therefore, represent a compensatory mechanism for the cells to sustain citrate production (Mullen et al, 2014). Alternatively, increased CS activity may be needed to support the biosynthetic requirements of PGC-1α–deficient cells by promoting citrate-derived fatty acid production and cell proliferation (MacPherson et al, 2017), which is consistent with the increased expression of PCNA at day 7.

Along with defective oxidative metabolism, the autophagic capacity of the RPE cells was severely altered after PGC-1α loss. Autophagic proteins are highly expressed in RPE cells and activation of the autophagic flux is essential for retinoid recycling and POS phagocytosis (Kim et al, 2013; Yao et al, 2014). As a direct target of the energy sensors and autophagy regulators AMPK and Sirt1, PGC-1α is also able to directly promote autophagy by increasing the expression of the lysosomal transcription factor TFEB (Tsunemi et al, 2012). Similarly, we found numerous genes involved in autophagosome assembly to be induced after PGC-1α overexpression, whereas PGC-1α deficiency repressed their expression, thereby blunting the autophagic flux and promoting the accumulation of defective organelles. Importantly, our investigation of the faulty autophagic step in PGC-1α–deficient RPE cells point to the inability of AMPK to be activated upon starvation, suggesting the existence of a positive feedback mechanism between AMPK and PGC-1α regulating RPE metabolism and autophagic functions.

A key finding from our in vitro and in vivo models is that PGC-1α deficiency ultimately promoted the loss of epithelial characteristics in RPE and induced EMT conversion. Metabolic reprogramming is emerging as a critical driver for the mesenchymal engagement of tumor cells, but its involvement in EMT of RPE cells is unknown. RPE epithelial status is tightly controlled by homotypic cell–cell interactions and growth factor signaling. Thus, loss of cell contact and exposure to pro-EMT extracellular stimuli such as TGF-β or TNF-α have been proposed as the main molecular mechanisms for promoting EMT of RPE (Takahashi et al, 2010). Here, we observed that loss of PGC-1α function is sufficient to induce RPE mesenchymal conversion independently of any exogenous stimuli. Collectively, our data suggest mitochondrial and autophagic deficiency as the underlying mechanisms for EMT in RPE secondary to PGC-1α silencing. First, the early perinuclear condensation of the vimentin IFs that we detected is likely a direct consequence of increased ROS as spatial reorganization of the vimentin network can be rapidly induced by increased cytosolic ROS production via the activation of the GTPase-activating protein Cdc42GAP (Li et al, 2009). However, true mesenchymal engagement of the cells was only observed at a later stage with the induction of TWIST1 and ZEB1, suggesting that sustained mitochondrial/autophagic dysfunction is necessary for the up-regulation of EMT-transcriptional factors. Recent evidence indicates that glucose metabolites can exert a profound effect on gene expression by epigenetic modifications. In stem cells, aerobic glycolysis sustains pluripotency by promoting acetyl-CoA production from pyruvate and increasing histone acetylation (Moussaieff et al, 2015) whereas, in liver cells, selective histone acetylation from lipid-derived acetyl-CoA specifically up-regulates genes involved in lipid metabolism (McDonnell et al, 2016). This illustrates that acetyl-CoA–dependent gene regulation is contextually controlled as histone acetylation at selective loci depends on both the metabolic source and spatiotemporal availability of acetyl-CoA (Campbell & Wellen, 2018). In the context of PGC-1α deficiency, the metabolic conversion of RPE from OXPHOS to glycolysis along with increased CS activity may also augment the acetyl-CoA pool allowing for histone acetylation and the activation of EMT-associated genes. Consistent with this hypothesis, histone deacetylase inhibitors increased EMT markers, including ZEB1, TWIST1, and vimentin in prostate cancer cells (Kong et al, 2012). In addition to transcriptionally inducing EMT-promoting genes, PGC-1α loss may also augment EMT protein levels. Indeed, a direct consequence of chronic deficient autophagic flux in our experimental cells is the accumulation of the autophagy substrate p62 shown to interact with TWIST1 protein to promote its stabilization and tumor cell proliferation (Qiang et al, 2014). Together, these findings indicate that PGC-1α–dependent regulation of glucose metabolism, metabolite shuttling, and autophagic flux in mature RPE cells is essential for the active repression of EMT and the maintenance of the cells’ epithelial status under basal conditions.

RPE dedifferentiation and EMT is involved in the onset and progression of common blinding ocular diseases, including AMD and PVR. Similar to the phenotypes we observed after conditional PGC-1 deletion in adult RPE, RPE from dry AMD patients are characterized by degradation of mitochondria, accumulation of lysosomes and increased mesenchymal markers (Ferrington et al, 2016; Golestaneh et al, 2016, 2017; Ghosh et al, 2018). Although the direct involvement of PGC-1α in AMD pathogenesis remains to be determined, it is interesting to note that AMD-like RPE dysmorphia with reduced autophagic capacity and increased p62 was also reported in global heterozygous PGC-1α+/− mice (Zhang et al, 2018).

In many pathological conditions, visual impairment from retinal degeneration is a direct consequence of RPE metabolic dysfunction and dedifferentiation. This can be seen in our in vivo data showing that initial dysfunction of RPE after deletion of PGC-1s (as indicated by their impaired light-evoked response) leads to progressive damage to neighboring cells as shown by the markedly reduced photoreceptor and bipolar cell ERG responses, culminating in a dramatic thinning of the retinal layers. Abnormalities in the *c*-wave amplitude and implicit time indicate defects in ion conductance across the RPE apical and basal membranes (Wu et al, 2004) and thus, suggest a potential role for metabolic regulation of RPE transepithelial potentials, an interesting and under-explored area for future studies.

Considering EMT is a plastic and revertible process, there is therapeutic potential for manipulation. Thus, promoting the re-epithelialization of mesenchymal RPE in the early stage of the disease through metabolic rewiring is an attractive strategy as similar approaches are already being investigated for the treatment of aggressive tumors (Morandi et al, 2017). As a key regulator of the main biological processes supporting RPE oxidative metabolism and repressing mesenchymal conversion, PGC-1α is emerging as an interesting therapeutic target for ocular diseases associated with RPE dystrophy.

## Materials and Methods

### Cell culture and shRNA-mediated PGC-1α gene knockdown in RPE

The human RPE cell line, ARPE-19 (CRL-2302; ATCC), was expanded in DMEM/F12 medium with 2 mM L-glutamine supplemented with 10% penicillin/streptomycin (Lonza) and 10% FBS (Atlanta Biologicals) at 37°C, 10% CO_2_. PGC-1α expression was silenced by lentivirus-mediated delivery of shRNAs selected from the Hannon-Ellege library (Silva et al, 2005) and cloned into a pGIPZ vector (Dharmacon). A pGIPZ nonsilencing shRNA (shControl) was used as negative control. To facilitate the delivery of shRNA into postmitotic cells, the pGIPZ-encoded shRNA was transfected into HEK293T cells along with a gag-pol-rev–containing packaging plasmid and an env-containing plasmid to pseudotype the viral capsids with the VSV-G (vesicular stomatitis virus glycoprotein) envelope protein (Dharmacon) with TransIT 293 transfection reagent (Thermo Fisher Scientific). Stably transduced GFP+ cells were selected by FACS sorting. For all cell assays, cells were dissociated with trypsin EDTA (Lonza) and counted with a Beckman Coulter (Beckman Coulter Brea) before plating. For maturation studies, confluent shPGC1A and shControl ARPE-19 cells were differentiated in serum-free condition for up to 21 d. For AAS, confluent cells were washed three times in Hank’s buffer (Gibco) and incubated for up to 24 h at 37°C in Earle’s Balanced Salt Solution (EBSS; Sigma-Aldrich) (Martina et al, 2016). For autophagic flux analysis, the cells were pretreated for 4 h with 40 μM CQ (Sigma-Aldrich) before AAS with EBSS.

### Western blotting analysis

Protein lysates were prepared using 1× cell lysis buffer (Cell Signaling) supplemented with 1 mM PMSF (Sigma-Aldrich) and quantified using the bicinchoninic acid method (Thermo Fisher Scientific Pierce). Total cell lysate (30 to 80 μg) was loaded into 10% SDS polyacrylamide gel (Bio-Rad). Proteins were transferred to PVDF membranes (Millipore). The membranes were blocked in 5% nonfat milk in TBS-T or PBS plus 0.1% Tween (PBS-T) for 1 h at RT. Membranes were incubated with primary antibodies, including anti-PGC-1α (1:250, #ST1202; Millipore); anti-LC3 (1:1,000, #3868); anti-LC3B (1:1,000, #2775), anti-PCNA (1:1,000, #13110), anti-SQSTM1/p62 (1:1,000, #88588), anti-total (1:500, #2532) and phospho-AMPKα Thr172 (1:250, #2531), anti-phospho-S6 Ser235/236 (1:1,000, #2211), and anti-α-tubulin (1:1,000, #3873), all from Cell Signaling Technology; anti-apolipoprotein E (1:1,000, #AB947; Chemicon International); anti-GAPDH (1:1,000, #SC25778), anti-actin (1:1,000, #2Q1055), anti-Twist (1:250, #SC-81417), and anti-ZEB1 (1:250, #SC-515797), from Santa Cruz Biotechnology. Subsequently, the membranes were incubated with the appropriate secondary antibodies and developed by chemiluminescence with SuperSignal West Pico Chemiluminescent Substrate (Thermo Fisher Scientific Pierce) or fluorescence method LI-COR Odyssey (LI-COR). Equal loading and transfer were ascertained by reprobing the membranes for α-tubulin, GAPDH, and actin. Exposed films were scanned and the densitometry analyses were performed by Image J2 (Rueden et al, 2017) or using Image Studio 2.0 (LI-COR). In some assays, arbitrary units of fluorescence were converted to fold changes.

### Immunocytochemistry

ARPE-19 cells were seeded at a concentration of 50,000 cells/cm^2^ on circle coverslips (12 mm of diameter). Once the cells reached 100% confluence, the complete medium was replaced by serum-free media for up to 21 d. Then, cells were washed with Hank's Balanced Salt Solution (Gibco), fixed with cold methanol (−20°C) for 10 min at RT and blocked with buffer solution (Hank's buffer, BSA 3% and 0.05% Tween) for 1 h. The cells were incubated with the appropriate primary antibodies: anti-ZO-1 (1:50, #61-7300; Invitrogen); anti-LAMP-1 (1:200, #9091; Cell Signaling Technology) and anti-Vimentin (1:200, #7783-500; Abcam), overnight at 4°C. The appropriate secondary antibodies were applied for 1 h at RT. Cell nuclei were stained with DAPI (Vector Laboratories INC). The slides were mounted with aqueous mounting medium (50% glycerol in PBS). The images were acquired with an Axioscope microscope (Carl Zeiss Microscopy). For mitochondria staining, unfixed cells were exposed to 100 nM MitoTracker Orange CMTMRos (Life Technologies) in Hank's buffer for 30 min at 37°C, 10% CO_2_. The ells were then washed, fixed with 4% paraformaldehyde in PBS, washed in Hank's buffer and permeabilized with 0.01% Triton X-100 (Sigma-Aldrich) for 5 min. Nuclei were stained using DAPI before mounting. Mitochondrial network images were acquired using an Axioscope microscope.

### Oxygen consumption rate and extracellular acidification rate measurement

Cells were plated at 50,000 cells/well in V7-PS microplates (Seahorse Bioscience) and matured for up to 21 d, For OCRs, the medium was replaced with the assay medium—minimal DMEM (Seahorse Bioscience) containing 2 mM glutamine (Lonza), 1 mM pyruvate (Gibco), and 25 mM glucose (Sigma-Aldrich), pH 7.4, and placed in a 37° CO_2_-free incubator for 30 min. OCR was assessed using an XF-24 Extracellular Flux Analyzer (Seahorse Biosciences), at baseline and after the sequential addition of 2.5 μM oligomycin (Sigma-Aldrich) to inhibit complex V, 500 nM carbonyl cyanide-4-(trifluoromethoxy) phenylhydrazone (FCCP) (Seahorse Bioscience) to uncouple the proton gradient, 2 μM rotenone and 2 μM antimycin A (Sigma-Aldrich) to inhibit complex I and III, respectively (Iacovelli et al, 2016). All OCR ([% Base Line][pMoles/min]) values reported were corrected to the number of cells per well quantified by DAPI staining (converted to fold change) and expressed as percentage. For extracellular acidification rate (ECAR), the medium was replaced with the assay medium—minimal DMEM (Seahorse Bioscience) containing 1 mM glutamine (pH 7.4) and placed in a 37° C CO_2_-free incubator for 1 h. ECAR was measured using an XF-24 Extracellular Flux Analyzer (Seahorse Biosciences) under basal conditions and after consecutive treatments with 10 mM glucose to induce glycolytic pathway, 2 μM oligomycin to promote glycolysis by blocking mitochondrial ATP production and 50 mM 2-deoxyglucose (2-DG) to block glycolysis. ECAR values (mpH/min) were normalized to the number of cells per well.

### ATP content and CSA

Total ATP levels were measured on cell lysates using the ATP bioluminescent assay CLS II kit (Roche) according to the manufacturer’s instructions. Values for each sample were normalized to the protein concentration. CSA was quantified in fresh protein samples (60 μg/ml) using the MitoCheck Citrate Synthase Activity Assay Kit (Cayman Chemicals) according to the manufacturer’s instructions.

### Quantification of mitochondrial superoxide generation and total reactive oxygen species

Cells (3 × 10^5^) were harvested and stained with 5 μM MitoSOX (Molecular Probes) or 10 μM CM-H_2_DCFDA (Life Technologies) at 37°C for 30 min to detect mitochondrial superoxide levels or total reactive oxygen species, respectively. The fluorescence intensity was analyzed using a fluorescence microplate reader (SynergyMx; Biotek) at excitation and emission wavelengths of 530 and 590 nm and 485 and 528 nm, respectively. For each assay, the arbitrary units of fluorescence from 0.33 × 10^5^ cells/well were averaged and converted to fold changes.

### Measurement of TER

Cells were seeded at 50,000 cells/well on laminin-coated 24-mm Transwell with 0.4-μm pore polyester membrane insert (Corning), left for 2 d to achieve confluency before maturation in serum-free medium for up to 21 d. TER was measured using an Epithelial Voltohmmeter (EVOM) with the STX100C electrode for 24-well format (World Precision Instruments). Resistance measurements in ohms (Ω) were calculated by subtracting the resistance of the filter alone (background) from the values obtained with the filters with RPE cells and expressed in Ω/cm^2^ (Rosales et al, 2014).

### Adenoviral infection

ARPE-19 were infected with control adenovirus (Ad-GFP; Vector Biolabs) or with adenovirus containing the mouse PGC-1α sequence (Ad-mPGC-1α, a gift of Dr. Arany, University of Pennsylvania, Philadelphia, PA) at a multiplicity of infection of 30 as previously described (Iacovelli et al, 2016). 24 h later, the cells were collected for RNA isolation. As PGC-1α functional domains are highly conserved across vertebrates (LeMoine et al, 2010), murine PGC-1α recapitulates the transcriptional functions of human PGC-1α (Diman et al, 2016).

### Animal studies and AAV injections

The animal work procedures complied with institutional animal care and authorizations in accordance with the local Committee for Ethics in Animal Research. Adult (8–12-wk old) C57Bl/6;PGC-1α^(fl/fl)^,PGC-1β^(fl/fl)^ male and female mice (generously provided by Dr. Arany, University of Pennsylvania, USA) were anesthetized by IP injection of ketamine and xylazine. Mydriasis was induced by topical application of 1% tropicamide. Right eyes were injected subretinally with 1 μl (2 × 10^9^ gc) of AAV2/8-CASI-GFP or AAV2/8-CASI-cre (as a mixture of 1.8 × 10^9^ gc AAV2/8-cre + 2 × 10^8^ gc AAV-GFP) using a 33G, point style three needle (Hamilton Company). 2 wk later, fundus imaging was performed to confirm transduction efficiency (GFP+) and lack of deleterious effects from the injection (subretinal hemorrhage, retinal detachment). Morphological and functional evaluations by fundus imaging, OCT, and ERG were performed at 1 and 4 mo postinjection. To control for potential Cre toxicity, 8-wk-old WT C57Bl/6 mice were subretinally injected with 2 × 10^9^ AAV2/8-cre or AAV2/8-GFP and evaluated as the experimental cohort by OCT, fundus photography, and ERG.

### Fundus photography

Fundus images were obtained on live anesthetized animals using the Micron III retinal imaging system (Phoenix Research Laboratories). The images were acquired using the bright-field and autofluorescent mode (488 nm excitation, 500–700 nm emission).

### OCT and retinal thickness quantification

OCT images centered on the optic nerve head were acquired on live anesthetized animals using the Bioptigen OCT system (Bioptigen). For each animal, full retinal thicknesses were measured at precisely 405 μm from the center of optic nerve head and averaged from 8 clock positions (2–8, 12–6, 11–5, and 9–4), where 12 o’clock is superior and 3 o’clock is nasal.

### ERG

Full-field ERG recordings were measured in dark-adapted and anesthetized animals using the Diagnosys ColorDome Ganzfeld ERG system (Diagnosys LLC). Recordings of the *a*- and *b*-waves were performed at light intensities of 0.00098–0.246 (P) cd.s/m^2^ and the *c*-wave response was measured as a flash intensity of 24.1 (P) cd.s/m^2^. The analyses were performed using Espion V6 Diagnosys Software. For each recording, the *a*-wave amplitude and implicit time was measured from the baseline to the trough of the first negative wave; the *b*-wave amplitude was measured from the trough of the *a*-wave to the peak of the highest positive wave and the implicit time from the baseline up to the highest positive wave; the *c*-wave amplitude and implicit time was measured from the baseline to the maximum recorded peak.

### Histology and electron microscopy

At 4 mo postinjection, mice were deeply anesthetized by injection of ketamine (62.5 mg/kg) and xylazine (12.5 mg/kg). Live animals were perfused via the aorta with 10 ml of sodium cacodylate buffer (0.1 M, pH 7.4) followed by 10 ml of ½ Karnovsky’s fixative in 0.1 M sodium cacodylate buffer (Electron Microscopy Sciences). Eyes were enucleated and the anterior segment removed. The eyecups were dehydrated and embedded in tEPON-812 epoxy resin. Semithin sections (1 μm) were stained with 1% toluidine blue in 1% sodium tetraborate aqueous solution for light microscopy. Ultrathin sections (80 nm) were cut from each sample block using a Leica EM UC7 ultramicrotome (Leica Microsystems) and stained with 2.5% aqueous gadolinium triacetate hydrate and Sato’s lead citrate stains using a modified Hiraoka grid staining system (Seifert, 2017). Grids were imaged using an FEI Tecnai G2 Spirit transmission electron microscope (FEI Company) at 80 kV interfaced with an AMT XR41 digital CCD camera (Advanced Microscopy Techniques) for digital TIFF file image acquisition. TEM imaging of retina samples were assessed and digital images captured at 2k×2k pixel, 16-bit resolution.

### Immunohistochemistry

Eyes were enucleated and fixed in 4% paraformaldehyde at 4°C overnight. RPE/choroid flat mounts were prepared by removal of the anterior segment and the retina. For cryosections, eyecups were prepared by removing the anterior segment and incubated in 30% sucrose overnight before embedding in Tissue-Tek OCT (Sakura Finetek) and cryosectioning (10 μm). RPE/choroid preparations and cryosections were blocked in 10% BSA, Triton 0.5% in PBS at RT for at least 1 h. Antimouse IgG fragment (0.12 mg/ml, #115-007-003; Jackson ImmunoResearch Laboratories) was added to the blocking buffer when using a mouse-raised primary antibody. The samples were then incubated overnight at 4°C with the following primary antibodies in blocking buffer: rabbit anti-Cre (1:100, #NB100-56133SS; Novus Biologicals), anti-GFP (1:200, #A-11122; Thermo Fisher Scientific), anti-ZO-1 (1:50, #61-7300) and Alexa 594–conjugated Phalloidin (1:100, #A-12381) both from Thermo/Invitrogen, rabbit anti-COX IV (1:200, #ab16056) and anti-Collagen VI (1:200, #ab6588) from Abcam; rabbit anti-Vimentin (1:200, #5741, Cell Signalling), and mouse anti-TWIST (1:250, #SC-81417) and ZEB-1 (1:250, #SC-515797) from Santa Cruz Biotechnology. After washing with PBS, the samples were incubated with a DyLight 549 goat anti-rabbit antibody (1:500; Vector Laboratories INC) or Alexa 594 anti-mouse antibody (1:500, #11062; Invitrogen) 1 h at RT. Cell nuclei were stained with DAPI (Vector). The slides were mounted with aqueous mounting medium (50% glycerol in PBS). The images were acquired with an Axioscope microscope (Carl Zeiss Microscopy). For quantification of mean fluorescence intensity, four to six randomly selected fields with flat RPE layout were imaged for each sample using identical exposure time. From each image, the median pixel intensity in the red channel was quantified using Adobe Photoshop CS6 software, and the results were averaged per sample.

### Real-time quantitative PCR analysis

Total mRNA from ARPE-19, RPE/choroid, and retina was isolated using RNA-Bee solution (IsoText Diagnostic Inc.). RNA was reverse-transcribed using the iScript kit (Bio-Rad). qPCR reactions were performed using the SYBR Green Master mix and the ABI Prism 9700 Sequence Detection System (Applied Biosystems) according to the manufacturer’s instructions, using specific primers (Table 1). Ct values were normalized to the housekeeping genes, *PPIA* and *HRPT1*, and relative changes in gene expression were calculated using the ΔΔCt method.

**Table 1.** Primer sequences for qPCR.

Gene symbol	Gene name	Forward sequence (5′-3′)	Reverse sequence (5′-3′)
Human primers			
*ATG4D*	Autophagy related 4D cysteine peptidase	CTCAACCCCGTGTATGTGC	TACAGTGAGTGTCGCGGTTT
*ATG9B*	Autophagy related 9B	GCTACTGGGACATCCAGGTG	AAGAGGCGGGACTGCAC
*CAT*	Catalase	ACTTTGAGGTCACACATGACATT	CTGAACCCGATTCTCCAGCA
*FIS1*	Mitochondrial fission 1 protein	TGACATCCGTAAAGGCATCG	CTTCTCGTATTCCTTGAGCCG
*GPX*	Glutathione peroxidase 1	CCAGTCGGTGTATGCCTTCTC	GAGGGACGCCACATTCTCG
*HMOX1*	Heme oxygenase 1	GCCAGCAACAAAGTGCAAG	GAGTGTAAGGACCCATCGGA
*HPRT1*	Hypoxanthine phosphoribosyltransferase 1	CCTGGCGTCGTGATTAGTGAT	AGACGTTCAGTCCTGTCCATAA
*LAMP1*	Lysosomal-associated membrane protein 1	ACGTTACAGCGTCCAGCTCAT	TCTTTGGAGCTCGCATTGG
*MAP1LC3B*	Microtubule associated protein 1 light chain 3 beta	GAGAAGACCTTCAAGCAGCG	TATCACCGGGATTTTGGTTG
*MCOLN1*	Mucolipin 1	TTGCTCTCTGCCAGCGGTACTA	GCAGTCAGTAACCACCATCGGA
*MFN2*	Mitofusin 2	ATGTGGCCCAACTCTAAGTG	CACAAACACATCAGCATCCAG
*PPARGC1A*	Peroxisome proliferator-activated receptor gamma, coactivator 1 alpha	AGCCTCTTTGCCCAGATCTT	CTGATTGGTCACTGCACCAC
*PPARGC1B*	Peroxisome proliferator-activated receptor gamma, coactivator 1 beta	CCACATCCTACCCAACATCAAG	CACAAGGCCGTTGACTTTTAGA
*POLG*	DNA polymerase gamma, catalytic subunit	GAAGGACATTCGTGAGAACTTCC	GTGGGGACACCTCTCCAAG
*PPIA*	Peptidylprolyl isomerase A	CAGACAAGGTCCCAAAGACAG	TTGCCATCCAACCACTCAGTC
*PRC*	PPARG related coactivator 1 (PPRC1)	GTGGTTGGGGAAGTCGAAG	TGACAAAGCCAGAATCACCC
*SOD1*	Superoxide dismutase 1, soluble	AGGGCATCATCAATTTCGAGC	GCCCACCGTGTTTTCTGGA
*SOD2*	Superoxide dismutase 2, mitochondrial	CAGACCTGCCTTACGACTATGG	CGTTCAGGTTGTTCACGTAGG
*SIRT1*	Sirtuin 1	TCAGTGTCATGGTTCCTTTGC	AATCTGCTCCTTTGCCACTCT
*SIRT3*	Sirtuin 3	TGGAAAGCCTAGTGGAGCTTCTGGG	TGGGGGCAGCCATCATCCTATTTGT
*TFAM*	Transcription factor a, mitochondrial	CCATATTTAAAGCTCAGAACCCAG	CTCCGCCCTATAAGCATCTTG
*TP53*	Tumor protein p53	TCAACAAGATGTTTTGCCAACTG	ATGTGCTGTGACTGCTTGTAGATG
*TXN2*	Thioredoxin 2	TGATGACCACACAGACCTCG	ATCCTTGATGCCCACAAACT
*TWIST1*	Twist family BHLH transcription factor 1	CGGAGAAGCTGAGCAAGATT	TGGAGGACCTGGTAGAGGAA
*VIM*	Vimentin	GAGAACTTTGCCGTTGAAGC	TCCAGCAGCTTCCTGTAGGT-3
*WIPI1*	WD repeat domain, phosphoinositide interacting 1	CCTCCTGGATATTCCTGCAA	GCACAATCTCCCCTGAAGTC
*ZEB1*	Zinc finger E-box binding homeobox 1	AGTGATCCAGCCAAATGGAA	TTTTTGGGCGGTGTAGAATC
*ZEB2*	Zinc finger E-box binding homeobox 2	AACAAGCCAATCCCAGGAG	GTTGGCAATACCGTCATCCT
Mouse primers			
*Hprt1*	Hypoxanthine phosphoribosyltransferase 1	TCAGTCAACGGGGGACATAAA	GGGGCTGTACTGCTTAACCAG
*Ppargc1a*	Peroxisome proliferator-activated receptor gamma, coactivator 1 alpha	AGCCGTGACCACTGACAACGAG	GTCGCATGGTTCTGAGTGCTAAG
*Ppargc1b*	Peroxisome proliferator-activated receptor gamma, coactivator 1 beta	CCCAGCGTCTGACGTGGACGAGC	CCTTCAGAGCGTCAGAGCTTGCTG
*Ppia*	Peptidylprolyl isomerase A	GAGCTGTTTGCAGACAAAGTTC	CCCTGGCACATGAATCCTGG

### Statistical analysis

Appropriate statistical tests were applied using GraphPad Prism 5.0 (GraphPad). In brief, *t* tests were used for comparisons between two groups. One-way ANOVA with Tukey post hoc test was used for comparison of three or more groups with one independent variable. Values were expressed as mean ± SEM. Significance was considered when *P* ≤ 0.05 and is indicated in the text as follows: *P < 0.05, **P < 0.01, ***P < 0.001, and ****P < 0.0001.

## Supplementary Material

Reviewer comments

## References

[bib1] Adijanto J, Castorino JJ, Wang ZX, Maminishkis A, Grunwald GB, Philp NJ (2012) Microphthalmia-associated transcription factor (MITF) promotes differentiation of human retinal pigment epithelium (RPE) by regulating microRNAs-204/211 expression. J Biol Chem 287: 20491–20503. 10.1074/jbc.m112.354761 22523078 PMC3370234

[bib2] Adijanto J, Philp NJ (2014) Cultured primary human fetal retinal pigment epithelium (hfRPE) as a model for evaluating RPE metabolism. Exp Eye Res 126: 77–84. 10.1016/j.exer.2014.01.015 24485945 PMC4345411

[bib3] Ahmad T, Aggarwal K, Pattnaik B, Mukherjee S, Sethi T, Tiwari BK, Kumar M, Micheal A, Mabalirajan U, Ghosh B, (2013) Computational classification of mitochondrial shapes reflects stress and redox state. Cell Death Dis 4: e461 10.1038/cddis.2012.213 23328668 PMC3564000

[bib4] Bertrand M, Petit V, Jain A, Amsellem R, Johansen T, Larue L, Codogno P, Beau I (2015) SQSTM1/p62 regulates the expression of junctional proteins through epithelial-mesenchymal transition factors. Cell Cycle 14: 364–374. 10.4161/15384101.2014.987619 25496309 PMC4353226

[bib5] Campbell SL, Wellen KE (2018) Metabolic signaling to the nucleus in cancer. Mol Cell 71: 398–408. 10.1016/j.molcel.2018.07.015 30075141

[bib6] Cantó C, Auwerx J (2009) PGC-1α, SIRT1 and AMPK, an energy sensing network that controls energy expenditure. Curr Opin Lipidol 20: 98–105. 10.1097/mol.0b013e328328d0a4 19276888 PMC3627054

[bib7] Casaroli–Marano RP, Pagan R, Vilaró S (1999) Epithelial–mesenchymal transition in proliferative vitreoretinopathy: Intermediate filament protein expression in retinal pigment epithelial cells. Invest Ophthalmol Vis Sci 40: 2062–2072. 10440262

[bib8] Chen M, Rajapakse D, Fraczek M, Luo C, Forrester JV, Xu H (2016) Retinal pigment epithelial cell multinucleation in the aging eye: A mechanism to repair damage and maintain homoeostasis. Aging Cell 15: 436–445. 10.1111/acel.12447 26875723 PMC4854907

[bib9] Demers-Lamarche J, Guillebaud G, Tlili M, Todkar K, Belanger N, Grondin M, Nguyen AP, Michel J, Germain M (2016) Loss of mitochondrial function impairs lysosomes. J Biol Chem 291: 10263–10276. 10.1074/jbc.m115.695825 26987902 PMC4858975

[bib10] Diman A, Boros J, Poulain F, Rodriguez J, Purnelle M, Episkopou H, Bertrand L, Francaux M, Deldicque L, Decottignies A (2016) Nuclear respiratory factor 1 and endurance exercise promote human telomere transcription. Sci Adv 2: e1600031 10.1126/sciadv.1600031 27819056 PMC5087959

[bib11] Du J, Yanagida A, Knight K, Engel AL, Vo AH, Jankowski C, Sadilek M, Tran VT, Manson MA, Ramakrishnan A, (2016) Reductive carboxylation is a major metabolic pathway in the retinal pigment epithelium. Proc Natl Acad Sci U S A 113: 14710–14715. 10.1073/pnas.1604572113 27911769 PMC5187684

[bib12] Egger A, Samardzija M, Sothilingam V, Tanimoto N, Lange C, Salatino S, Fang L, Garcia-Garrido M, Beck S, Okoniewski MJ, (2012) PGC-1alpha determines light damage susceptibility of the murine retina. PLoS One 7: e31272 10.1371/journal.pone.0031272 22348062 PMC3278422

[bib13] Felszeghy S, Viiri J, Paterno JJ, Hyttinen JMT, Koskela A, Chen M, Leinonen H, Tanila H, Kivinen N, Koistinen A, (2019) Loss of NRF-2 and PGC-1α genes leads to retinal pigment epithelium damage resembling dry age-related macular degeneration. Redox Biol 20: 1–12. 10.1016/j.redox.2018.09.011 30253279 PMC6156745

[bib14] Fernandez-Marcos PJ, Auwerx J (2011) Regulation of PGC-1alpha, a nodal regulator of mitochondrial biogenesis. Am J Clin Nutr 93: 884S–890S. 10.3945/ajcn.110.001917 21289221 PMC3057551

[bib15] Ferrington DA, Ebeling MC, Kapphahn RJ, Terluk MR, Fisher CR, Polanco JR, Roehrich H, Leary MM, Geng Z, Dutton JR, (2017) Altered bioenergetics and enhanced resistance to oxidative stress in human retinal pigment epithelial cells from donors with age-related macular degeneration. Redox Biol 13: 255–265. 10.1016/j.redox.2017.05.015 28600982 PMC5466586

[bib16] Ferrington DA, Sinha D, Kaarniranta K (2016) Defects in retinal pigment epithelial cell proteolysis and the pathology associated with age-related macular degeneration. Prog Retin Eye Res 51: 69–89. 10.1016/j.preteyeres.2015.09.002 26344735 PMC4769684

[bib17] Ghosh S, Shang P, Terasaki H, Stepicheva N, Hose S, Yazdankhah M, Weiss J, Sakamoto T, Bhutto IA, Xia S, (2018) A role for betaA3/A1-crystallin in type 2 EMT of RPE cells occurring in dry age-related macular degeneration. Invest Ophthalmol Vis Sci 59: AMD104–AMD113. 10.1167/iovs.18-24132 30098172 PMC6058694

[bib18] Golestaneh N, Chu Y, Cheng SK, Cao H, Poliakov E, Berinstein DM (2016) Repressed SIRT1/PGC-1alpha pathway and mitochondrial disintegration in iPSC-derived RPE disease model of age-related macular degeneration. J Transl Med 14: 344 10.1186/s12967-016-1101-8 27998274 PMC5175395

[bib19] Golestaneh N, Chu Y, Xiao YY, Stoleru GL, Theos AC (2017) Dysfunctional autophagy in RPE, a contributing factor in age-related macular degeneration. Cell Death Dis 8: e2537 10.1038/cddis.2016.453 28055007 PMC5386365

[bib20] Grassi G, Di Caprio G, Santangelo L, Fimia GM, Cozzolino AM, Komatsu M, Ippolito G, Tripodi M, Alonzi T (2015) Autophagy regulates hepatocyte identity and epithelial-to-mesenchymal and mesenchymal-to-epithelial transitions promoting Snail degradation. Cell Death Dis 6: e1880 10.1038/cddis.2015.249 26355343 PMC4650445

[bib21] Guerra F, Guaragnella N, Arbini AA, Bucci C, Giannattasio S, Moro L (2017) Mitochondrial dysfunction: A novel potential driver of epithelial-to-mesenchymal transition in cancer. Front Oncol 7: 295 10.3389/fonc.2017.00295 29250487 PMC5716985

[bib22] Gugnoni M, Sancisi V, Manzotti G, Gandolfi G, Ciarrocchi A (2016) Autophagy and epithelial-mesenchymal transition: An intricate interplay in cancer. Cell Death Dis 7: e2520 10.1038/cddis.2016.415 27929542 PMC5260980

[bib23] Hirasawa M, Noda K, Noda S, Suzuki M, Ozawa Y, Shinoda K, Inoue M, Ogawa Y, Tsubota K, Ishida S (2011) Transcriptional factors associated with epithelial-mesenchymal transition in choroidal neovascularization. Mol Vis 17: 1222–1230. 21617757 PMC3102030

[bib24] Iacovelli J, Rowe GC, Khadka A, Diaz-Aguilar D, Spencer C, Arany Z, Saint-Geniez M (2016) PGC-1alpha induces human RPE oxidative metabolism and antioxidant capacity. Invest Ophthalmol Vis Sci 57: 1038–1051. 10.1167/iovs.15-17758 26962700 PMC4788093

[bib25] Jiang M, Esteve-Rudd J, Lopes VS, Diemer T, Lillo C, Rump A, Williams DS (2015) Microtubule motors transport phagosomes in the RPE, and lack of KLC1 leads to AMD-like pathogenesis. J Cell Biol 210: 595–611. 10.1083/jcb.201410112 26261180 PMC4539993

[bib26] Kaarniranta K, Kajdanek J, Morawiec J, Pawlowska E, Blasiak J (2018) PGC-1alpha protects RPE cells of the aging retina against oxidative stress-induced degeneration through the regulation of senescence and mitochondrial quality control. The significance for AMD pathogenesis. Int J Mol Sci 19: E2317 10.3390/ijms19082317 30087287 PMC6121367

[bib27] Kaarniranta K, Sinha D, Blasiak J, Kauppinen A, Vereb Z, Salminen A, Boulton ME, Petrovski G (2013) Autophagy and heterophagy dysregulation leads to retinal pigment epithelium dysfunction and development of age-related macular degeneration. Autophagy 9: 973–984. 10.4161/auto.24546 23590900 PMC3722332

[bib28] Kim JY, Zhao H, Martinez J, Doggett TA, Kolesnikov AV, Tang PH, Ablonczy Z, Chan CC, Zhou Z, Green DR, (2013) Noncanonical autophagy promotes the visual cycle. Cell 154: 365–376. 10.1016/j.cell.2013.06.012 23870125 PMC3744125

[bib29] Kirkland RA, Franklin JL (2001) Evidence for redox regulation of cytochrome C release during programmed neuronal death: Antioxidant effects of protein synthesis and caspase inhibition. J Neurosci 21: 1949–1963. 10.1523/jneurosci.21-06-01949.2001 11245680 PMC6762616

[bib30] Kong D, Ahmad A, Bao B, Li Y, Banerjee S, Sarkar FH (2012) Histone deacetylase inhibitors induce epithelial-to-mesenchymal transition in prostate cancer cells. PLoS One 7: e45045 10.1371/journal.pone.0045045 23024790 PMC3443231

[bib31] Kong X, Wang R, Xue Y, Liu X, Zhang H, Chen Y, Fang F, Chang Y (2010) Sirtuin 3, a new target of PGC-1alpha, plays an important role in the suppression of ROS and mitochondrial biogenesis. PLoS One 5: e11707 10.1371/journal.pone.0011707 20661474 PMC2908542

[bib32] LeMoine CM, Lougheed SC, Moyes CD (2010) Modular evolution of PGC-1alpha in vertebrates. J Mol Evol 70: 492–505. 10.1007/s00239-010-9347-x 20443112

[bib33] Li QF, Spinelli AM, Tang DD (2009) Cdc42GAP, reactive oxygen species, and the vimentin network. Am J Physiol Cell Physiol 297: C299–C309. 10.1152/ajpcell.00037.2009 19494238 PMC2724092

[bib34] Lin H, Xu H, Liang FQ, Liang H, Gupta P, Havey AN, Boulton ME, Godley BF (2011) Mitochondrial DNA damage and repair in RPE associated with aging and age-related macular degeneration. Invest Ophthalmol Vis Sci 52: 3521–3529. 10.1167/iovs.10-6163 21273542 PMC3109040

[bib35] Machemer R, van Horn D, Aaberg TM (1978) Pigment epithelial proliferation in human retinal detachment with massive periretinal proliferation. Am J Ophthalmol 85: 181–191. 10.1016/s0002-9394(14)75946-x 623188

[bib36] MacPherson S, Horkoff M, Gravel C, Hoffmann T, Zuber J, Lum JJ (2017) STAT3 regulation of citrate synthase is essential during the initiation of lymphocyte cell growth. Cell Rep 19: 910–918. 10.1016/j.celrep.2017.04.012 28467904

[bib37] Martina JA, Diab HI, Brady OA, Puertollano R (2016) TFEB and TFE3 are novel components of the integrated stress response. EMBO J 35: 479–495. 10.15252/embj.201593428 26813791 PMC4772850

[bib38] McDonnell E, Crown SB, Fox DB, Kitir B, Ilkayeva OR, Olsen CA, Grimsrud PA, Hirschey MD (2016) Lipids reprogram metabolism to become a major carbon source for histone acetylation. Cell Rep 17: 1463–1472. 10.1016/j.celrep.2016.10.012 27806287 PMC5123807

[bib39] Morandi A, Taddei ML, Chiarugi P, Giannoni E (2017) Targeting the metabolic reprogramming that controls epithelial-to-mesenchymal transition in aggressive tumors. Front Oncol 7: 40 10.3389/fonc.2017.00040 28352611 PMC5348536

[bib40] Moussaieff A, Rouleau M, Kitsberg D, Cohen M, Levy G, Barasch D, Nemirovski A, Shen-Orr S, Laevsky I, Amit M, (2015) Glycolysis-mediated changes in acetyl-CoA and histone acetylation control the early differentiation of embryonic stem cells. Cell Metab 21: 392–402. 10.1016/j.cmet.2015.02.002 25738455

[bib41] Mullen AR, Hu Z, Shi X, Jiang L, Boroughs LK, Kovacs Z, Boriack R, Rakheja D, Sullivan LB, Linehan WM, (2014) Oxidation of alpha-ketoglutarate is required for reductive carboxylation in cancer cells with mitochondrial defects. Cell Rep 7: 1679–1690. 10.1016/j.celrep.2014.04.037 24857658 PMC4057960

[bib42] Osellame LD, Rahim AA, Hargreaves IP, Gegg ME, Richard-Londt A, Brandner S, Waddington SN, Schapira AH, Duchen MR (2013) Mitochondria and quality control defects in a mouse model of Gaucher disease: Links to Parkinson’s disease. Cell Metab 17: 941–953. 10.1016/j.cmet.2013.04.014 23707074 PMC3678026

[bib43] Qiang L, Zhao B, Ming M, Wang N, He TC, Hwang S, Thorburn A, He YY (2014) Regulation of cell proliferation and migration by p62 through stabilization of Twist1. Proc Natl Acad Sci U S A 111: 9241–9246. 10.1073/pnas.1322913111 24927592 PMC4078859

[bib44] Roggia MF, Ueta T (2015) alphavbeta5 integrin/FAK/PGC-1alpha pathway confers protective effects on retinal pigment epithelium. PLoS One 10: e0134870 10.1371/journal.pone.0134870 26244551 PMC4526642

[bib45] Rosales MAB, Silva KC, Duarte DA, Rossato FA, Lopes de Faria JB, Lopes de Faria JM (2014) Endocytosis of tight junctions caveolin nitrosylation dependent is improved by cocoa via opioid receptor on RPE cells in diabetic conditions. Invest Ophthalmol Vis Sci 55: 6090–6100. 10.1167/iovs.14-14234 25190662

[bib46] Rowe GC, Patten IS, Zsengeller ZK, El-Khoury R, Okutsu M, Bampoh S, Koulisis N, Farrell C, Hirshman MF, Yan Z, (2013) Disconnecting mitochondrial content from respiratory chain capacity in PGC-1-deficient skeletal muscle. Cell Rep 3: 1449–1456. 10.1016/j.celrep.2013.04.023 23707060 PMC3688451

[bib47] Rueden CT, Schindelin J, Hiner MC, DeZonia BE, Walter AE, Arena ET, Eliceiri KW (2017) ImageJ2: ImageJ for the next generation of scientific image data. BMC Bioinformatics 18: 529 10.1186/s12859-017-1934-z 29187165 PMC5708080

[bib48] Saadat KA, Murakami Y, Tan X, Nomura Y, Yasukawa T, Okada E, Ikeda Y, Yanagi Y (2014) Inhibition of autophagy induces retinal pigment epithelial cell damage by the lipofuscin fluorophore A2E. FEBS Open Bio 4: 1007–1014. 10.1016/j.fob.2014.11.003 PMC4250541 25473597

[bib65] Satish S, Philipose H, Rosales MAB, Saint-Geniez M (2018) Pharmaceutical Induction of PGC-1*α* Promotes Retinal Pigment Epithelial Cell Metabolism and Protects against Oxidative Damage. Oxid Med Cell Longev 2018: 9248640 10.1155/2018/9248640 30524663 PMC6247391

[bib49] Schlichtholz B, Turyn J, Goyke E, Biernacki M, Jaskiewicz K, Sledzinski Z, Swierczynski J (2005) Enhanced citrate synthase activity in human pancreatic cancer. Pancreas 30: 99–104. 10.1097/01.mpa.0000153326.69816.7d 15714131

[bib50] Sczelecki S, Besse-Patin A, Abboud A, Kleiner S, Laznik-Bogoslavski D, Wrann CD, Ruas JL, Haibe-Kains B, Estall JL (2014) Loss of Pgc-1alpha expression in aging mouse muscle potentiates glucose intolerance and systemic inflammation. Am J Physiol Endocrinol Metab 306: E157–E167. 10.1152/ajpendo.00578.2013 24280126 PMC4073996

[bib51] Seifert P (2017) Modified Hiraoka TEM grid staining apparatus and technique using 3D printed materials and gadolinium triacetate tetrahydrate, a nonradioactive uranyl acetate substitute. J Histotechnol 40: 130–135. 10.1080/01478885.2017.1361117 31105361 PMC6519938

[bib66] Silva JM, Li MZ, Chang K, Ge W, Golding MC, Rickles RJ, Siolas D, Hu G, Paddison PJ, Schlabach MR, (2005) Second-generation shRNA libraries covering the mouse and human genomes. Nat. Genet 37: 1281–8. 10.1038/ng1650 16200065

[bib52] Sripathi SR, He W, Um JY, Moser T, Dehnbostel S, Kindt K, Goldman J, Frost MC, Jahng WJ (2012) Nitric oxide leads to cytoskeletal reorganization in the retinal pigment epithelium under oxidative stress. Adv Biosci Biotechnol 3: 1167–1178. 10.4236/abb.2012.38143 27974994 PMC5152770

[bib53] Steen L, Joachim N, Neigaard HC, Bo NL, Flemming W, Nis S, Daa SH, Robert B, Wulff HJ, Flemming D, (2012) Biomarkers of mitochondrial content in skeletal muscle of healthy young human subjects. The J Physiol 590: 3349–3360. 22586215 10.1113/jphysiol.2012.230185 PMC3459047

[bib54] Strauss O (2005) The retinal pigment epithelium in visual function. Physiol Rev 85: 845–881. 10.1152/physrev.00021.2004 15987797

[bib55] Takahashi E, Nagano O, Ishimoto T, Yae T, Suzuki Y, Shinoda T, Nakamura S, Niwa S, Ikeda S, Koga H, (2010) Tumor necrosis factor-alpha regulates transforming growth factor-beta-dependent epithelial-mesenchymal transition by promoting hyaluronan-CD44-moesin interaction. J Biol Chem 285: 4060–4073. 10.1074/jbc.m109.056523 19965872 PMC2823547

[bib56] Tsunemi T, Ashe TD, Morrison BE, Soriano KR, Au J, Roque RA, Lazarowski ER, Damian VA, Masliah E, La Spada AR (2012) PGC-1alpha rescues Huntington’s disease proteotoxicity by preventing oxidative stress and promoting TFEB function. Sci Transl Med 4: 142ra197 10.1126/scitranslmed.3003799 PMC4096245 22786682

[bib57] Vainshtein A, Tryon LD, Pauly M, Hood DA (2015) Role of PGC-1alpha during acute exercise-induced autophagy and mitophagy in skeletal muscle. Am J Physiol Cell Physiol 308: C710–C719. 10.1152/ajpcell.00380.2014 25673772 PMC4420796

[bib58] Vandenberghe LH, Bell P, Maguire AM, Cearley CN, Xiao R, Calcedo R, Wang L, Castle MJ, Maguire AC, Grant R, (2011) Dosage thresholds for AAV2 and AAV8 photoreceptor gene therapy in monkey. Sci Transl Med 3: 88ra54 10.1126/scitranslmed.3002103 PMC5027886 21697530

[bib59] Vishnyakova PA, Volodina MA, Tarasova NV, Marey MV, Tsvirkun DV, Vavina OV, Khodzhaeva ZS, Kan NE, Menon R, Vysokikh MY, (2016) Mitochondrial role in adaptive response to stress conditions in preeclampsia. Scientific Rep 6: 32410 10.1038/srep32410 PMC5004102 27573305

[bib60] Wu J, Peachey NS, Marmorstein AD (2004) Light-evoked responses of the mouse retinal pigment epithelium. J Neurophysiol 91: 1134–1142. 10.1152/jn.00958.2003 14614107 PMC2897140

[bib61] Wu Z, Puigserver P, Andersson U, Zhang C, Adelmant G, Mootha V, Troy A, Cinti S, Lowell B, Scarpulla RC, (1999) Mechanisms controlling mitochondrial biogenesis and respiration through the thermogenic coactivator PGC-1. Cell 98: 115–124. 10.1016/s0092-8674(00)80611-x 10412986

[bib62] Yao J, Jia L, Shelby SJ, Ganios AM, Feathers K, Thompson DA, Zacks DN (2014) Circadian and noncircadian modulation of autophagy in photoreceptors and retinal pigment epithelium. Invest Ophthalmol Vis Sci 55: 3237–3246. 10.1167/iovs.13-13336 24781939 PMC4037936

[bib63] Zhang J, Bai Y, Huang L, Qi Y, Zhang Q, Li S, Wu Y, Li X (2015) Protective effect of autophagy on human retinal pigment epithelial cells against lipofuscin fluorophore A2E: Implications for age-related macular degeneration. Cell Death Dis 6: e1972 10.1038/cddis.2015.330 26561782 PMC4670934

[bib64] Zhang M, Chu Y, Mowery J, Konkel B, Galli S, Theos AC, Golestaneh N (2018) Pgc-1alpha repression and high-fat diet induce age-related macular degeneration-like phenotypes in mice. Dis Model Mech 11: dmm032698 10.1242/dmm.032698 29925537 PMC6176989

