# Correction: Loss of PGC-1α in RPE induces mesenchymal transition and promotes retinal degeneration

**DOI:** 10.26508/lsa.201900436

**Published:** 2019-05-29

**Authors:** Mariana Aparecida Brunini Rosales, Daisy Y Shu, Jared Iacovelli, Magali Saint-Geniez

**Affiliations:** 1 Schepens Eye Research Institute of Massachusetts Eye and Ear, Boston, MA; 2 Department of Ophthalmology, Harvard Medical School, Boston, MA

Following publication, the authors realized that Table 1 inadvertently missed some primer sequences and contained primer sequences for genes not tested in this study. The table has now been replaced with the correct one. The authors regret any confusion this may have caused. Both the HTML and PDF versions of the article have been corrected. The corrected table is given below.

**Table 1.** Primer sequences for qPCR.

Gene symbol	Gene name	Forward sequence (5′-3′)	Reverse sequence (5′-3′)
Human primers			
*ATG4D*	Autophagy related 4D cysteine peptidase	CTCAACCCCGTGTATGTGC	TACAGTGAGTGTCGCGGTTT
*ATG9B*	Autophagy related 9B	GCTACTGGGACATCCAGGTG	AAGAGGCGGGACTGCAC
*CAT*	Catalase	ACTTTGAGGTCACACATGACATT	CTGAACCCGATTCTCCAGCA
*FIS1*	Mitochondrial fission 1 protein	TGACATCCGTAAAGGCATCG	CTTCTCGTATTCCTTGAGCCG
*GPX*	Glutathione peroxidase 1	CCAGTCGGTGTATGCCTTCTC	GAGGGACGCCACATTCTCG
*HMOX1*	Heme oxygenase 1	GCCAGCAACAAAGTGCAAG	GAGTGTAAGGACCCATCGGA
*HPRT1*	Hypoxanthine phosphoribosyltransferase 1	CCTGGCGTCGTGATTAGTGAT	AGACGTTCAGTCCTGTCCATAA
*LAMP1*	Lysosomal-associated membrane protein 1	ACGTTACAGCGTCCAGCTCAT	TCTTTGGAGCTCGCATTGG
*MAP1LC3B*	Microtubule associated protein 1 light chain 3 beta	GAGAAGACCTTCAAGCAGCG	TATCACCGGGATTTTGGTTG
*MCOLN1*	Mucolipin 1	TTGCTCTCTGCCAGCGGTACTA	GCAGTCAGTAACCACCATCGGA
*MFN2*	Mitofusin 2	ATGTGGCCCAACTCTAAGTG	CACAAACACATCAGCATCCAG
*PPARGC1A*	Peroxisome proliferator-activated receptor gamma, coactivator 1 alpha	AGCCTCTTTGCCCAGATCTT	CTGATTGGTCACTGCACCAC
*PPARGC1B*	Peroxisome proliferator-activated receptor gamma, coactivator 1 beta	CCACATCCTACCCAACATCAAG	CACAAGGCCGTTGACTTTTAGA
*POLG*	DNA polymerase gamma, catalytic subunit	GAAGGACATTCGTGAGAACTTCC	GTGGGGACACCTCTCCAAG
*PPIA*	Peptidylprolyl isomerase A	CAGACAAGGTCCCAAAGACAG	TTGCCATCCAACCACTCAGTC
*PRC*	PPARG related coactivator 1 (PPRC1)	GTGGTTGGGGAAGTCGAAG	TGACAAAGCCAGAATCACCC
*SOD1*	Superoxide dismutase 1, soluble	AGGGCATCATCAATTTCGAGC	GCCCACCGTGTTTTCTGGA
*SOD2*	Superoxide dismutase 2, mitochondrial	CAGACCTGCCTTACGACTATGG	CGTTCAGGTTGTTCACGTAGG
*SIRT1*	Sirtuin 1	TCAGTGTCATGGTTCCTTTGC	AATCTGCTCCTTTGCCACTCT
*SIRT3*	Sirtuin 3	TGGAAAGCCTAGTGGAGCTTCTGGG	TGGGGGCAGCCATCATCCTATTTGT
*TFAM*	Transcription factor a, mitochondrial	CCATATTTAAAGCTCAGAACCCAG	CTCCGCCCTATAAGCATCTTG
*TP53*	Tumor protein p53	TCAACAAGATGTTTTGCCAACTG	ATGTGCTGTGACTGCTTGTAGATG
*TXN2*	Thioredoxin 2	TGATGACCACACAGACCTCG	ATCCTTGATGCCCACAAACT
*TWIST1*	Twist family BHLH transcription factor 1	CGGAGAAGCTGAGCAAGATT	TGGAGGACCTGGTAGAGGAA
*VIM*	Vimentin	GAGAACTTTGCCGTTGAAGC	TCCAGCAGCTTCCTGTAGGT-3
*WIPI1*	WD repeat domain, phosphoinositide interacting 1	CCTCCTGGATATTCCTGCAA	GCACAATCTCCCCTGAAGTC
*ZEB1*	Zinc finger E-box binding homeobox 1	AGTGATCCAGCCAAATGGAA	TTTTTGGGCGGTGTAGAATC
*ZEB2*	Zinc finger E-box binding homeobox 2	AACAAGCCAATCCCAGGAG	GTTGGCAATACCGTCATCCT
Mouse primers			
*Hprt1*	Hypoxanthine phosphoribosyltransferase 1	TCAGTCAACGGGGGACATAAA	GGGGCTGTACTGCTTAACCAG
*Ppargc1a*	Peroxisome proliferator-activated receptor gamma, coactivator 1 alpha	AGCCGTGACCACTGACAACGAG	GTCGCATGGTTCTGAGTGCTAAG
*Ppargc1b*	Peroxisome proliferator-activated receptor gamma, coactivator 1 beta	CCCAGCGTCTGACGTGGACGAGC	CCTTCAGAGCGTCAGAGCTTGCTG
*Ppia*	Peptidylprolyl isomerase A	GAGCTGTTTGCAGACAAAGTTC	CCCTGGCACATGAATCCTGG

